# A lectin gene is involved in the defense of *Pleurotus ostreatus* against the mite predator *Tyrophagus putrescentiae*

**DOI:** 10.3389/fmicb.2023.1191500

**Published:** 2023-04-27

**Authors:** Junjie Liu, Huiping Li, Xin Luo, Lin Ma, Cuixin Li, Shaoxuan Qu

**Affiliations:** ^1^School of Life Sciences, Southwest Forestry University, Kunming, Yunnan, China; ^2^Institute of Vegetable Crops, Jiangsu Academy of Agricultural Sciences, Nanjing, China

**Keywords:** fungal lectin, edible fungi, the storage mite, fungal defense, oyster mushroom

## Abstract

The storage mite, *Tyrophagus putrescentiae*, found worldwide in many habitats, is an important pest of edible mushrooms. Excessive chemical spraying for pest control has been linked to environmental pollution, health risks, insecticide resistance development, and food safety. Host resistance can be sustainable and cost-effective and provide effective and economical pest control. Previous studies have reported that the oyster mushroom *Pleurotus ostreatus* has evolved effective defense mechanisms against *T. putrescentiae* attack, but the underlying mechanism remains unclear. Here we report that a lectin gene from *P. ostreatus* mycelia, *Polec2*, induced fungal resistance to mite grazing. *Polec2* belongs to a galectin-like lectin classification, encoding a protein with β-sandwith-fold domain. Overexpression of *Polec2* in *P. ostreatus* led to activation of the reactive oxygen species (ROS)/mitogen-activated protein kinases (MAPKs) signaling pathway, salicylic acid (SA), and jasmonate (JA) biosynthesis. The activation resulted in bursts of antioxidant activities of catalases (CAT), peroxidases (POD), superoxide dismutases (SOD), and increased production of SA, JA, jasmonic acid-isoleucine (JA-Ile) and jasmonic acid methyl ester (MeJA), accompanied by reduced *T. putrescentiae* feeding and suppressed its population. We also provide an overview of the phylogenetic distribution of lectins across 22 fungal genomes. Our findings shed light on the molecular mechanisms of *P. ostreatus*’ defense against the mite predator and will be useful in investigating the molecular basis of fungi-fungivory interactions and gene mining for pest-resistance genes.

## 1. Introduction

Organisms have evolved efficient defense mechanisms to protect themselves from biotic and abiotic stresses (Naranjo-Ortiz and Gabaldón, 2019). Innate immunity plays a critical role, and hence con-structs the first defensive line, in animals and pathogens. In modern agriculture, cultivating crop varieties with durable and broad-spectrum resistance is an effective strategy (Li et al., 2003; Dangl et al., 2013; Deng et al., 2017). Currently, pests of edible mushrooms are primarily controlled with pesticides, leading to pest resistance problems, pesticide residues, and food safety risks (Wang et al., 2016). Hence, innate immunity can be used to improve edible mushrooms’ recalcitrance to pest attacks (Arthur et al., 2014).

Edible mushroom hosts affect the feeding preference, reproduction, longevity, and life table parameters of pests (Kunzler, 2015). The different cultivars of the oyster mushroom *Pleurotus ostreatus* have a significant effect on the biological parameters of the storage mite, *Tyrophagus putrescentiae*, whose infestation reduces mushroom yield (Qu et al., 2015; Hou et al., 2022). Previous studies have reported that plant nutrients impact insect herbivore performance (Wetzel et al., 2016). In our recent work, *P. ostreatus* was shown to have developed several defense systems against *T. putrescentiae* feeding (Li et al., 2022). Fungus *P. ostreatus* coordinated inducible chemical-based defense responses through the reactive oxygen species (ROS)/mitogen-activated protein kinases (MAPKs) signaling pathway, jasmonate (JA) regulation, specific gene expression and protein synthesis, and anti-mite substance metabolism. Furthermore, upregulated genes were related to terpenoid metabolism, including the P450 family genes and those encoding toxins, such as lectins (Li et al., 2022). Fungal volatile terpenes play an important role in the communications of fungus-mite interactions (Li et al., 2018).

Lectins are a group of carbohydrate-binding proteins that can specifically bind polysaccharides, glycoproteins, and glycolipids on cell surfaces (Ji and Nicolson, 1974). Lectins can recognize exogenous carbohydrates and often exhibit anti-competitor, anti-predator, anti-parasite, and anti-pathogen activities. Mushrooms are rich in lectins. Fungi have massive hemagglutinins (early terminology for lectins). Lectins purified from *Gymnopilus* mushrooms were shown to inhibit *Staphylococcus aureus* and *Aspergillus niger*, suggesting a role for lectin in defense (Alborés et al., 2014; Singh et al., 2020).

In earlier research, lectin proteins AAL, MOA, and TAP1 purified through *in vitro* expression from *Aleuria aurantia*, *Marasmius oreades*, and *Sordaria macrospora*, respectively, demonstrated toxicity against *Aphelenchus avenae* and *Bursaphelenchus okinawaensis*. However, CCL2 and CGL2, which are toxic to the nematode *Caenorhabditis elegans*, did not exhibit the same effect on *A. avenae* (Butschi et al., 2010; Tayyrov et al., 2018; Singh et al., 2020). It is also proposed that lectins are involved in the biological activities of mushrooms, including participation in fungal growth, development, mycorrhiza formation, and defense against predators and parasites (Kawagishi et al., 1997). Previous studies have revealed a linkage of lectins and hemolysins to why *Lepista* and *Cantharellus* mushrooms barely attract insects (Pohleven et al., 2011; Varrot et al., 2013). Lectins are mainly enriched in mushrooms’ fruit bodies and sclerotia (Wang et al., 2002). Most of them are expressed in the cytoplasm, but cytoplasmic ligands deficiency makes it hard to correspond with endogenous function (Wohlschlager et al., 2014). Meanwhile, the absence of lectins does not affect fungal development (either by knockout or knockdown), demonstrating that its function is exogenous. Massive structural lectin families from fungal fruit bodies have been characterized, mainly galectin, β-trefoil-type, β-propeller-type, actinoporin-type, cyanovirin-N-type, and immunoglobulin-type. Some of these have been reported to have insecticidal properties (Bleuler-Martínez et al., 2011; Plaza et al., 2015; Bleuler-Martinez et al., 2017; Lebreton et al., 2021; Bleuler-Martinez et al., 2022). Lectins abundant in *P. ostreatus* have been widely reported for their anti-tumor activity (Vajravijayan et al., 2020). Crystals of *P. ostreatus* lectin was first reported with a molecular mass of 293 kDa (Chattopadhyay et al., 1999). Then, the structure and function of a *P. ostreatus* lectin named POL is reported that the protein active site requires two adjacent alcohol groups, one in the axial position of the sugar and the other in the equatorial position of the sugar, adjacent to which a calcium-mediated binding loop can be identified (Vajravijayan et al., 2020). Interestingly, a lectin from *P. ostreatus* named *Plp* has been found to be induced by blue light (Perduca et al., 2020). However, *P. ostreatus* anti-predator lectins remain understood limitedly.

In this work, we investigated the defense mechanism of an anti-predator lectin gene, named *Polec2,* which was significantly up-regulated in *P. ostreatus* mycelium after *T. putrescentiae* feeding (Li et al., 2022). To gain an insight into the molecular and biochemical properties of the oyster mushroom against *T. putrescentiae*, we examined effect of *Polec2* overexpression on the mushroom’s defense system. The characteristics of this lectin were compared with those anti-predator lectins characterized previously. These findings unveil an important anti-predator activity of *Polec2* and expand a basis of the mushroom’s resistance to *T. putrescentiae*.

## 2. Materials and methods

### 2.1. Strains and culture conditions

The dikaryotic *P. osteatus* strain CCMSSC00389 (China Center for Mushroom Spawn Standards and Control, CCMSSC; designated WT hereafter) was grown on potato dextrose agar (PDA) at 25°C under darkness for 7 days. For RNA isolation and RNA Sequencing, the vegetative mycelia of the strain were collected after cultivation in 100 ml PDB (potato dextrose broth) in the dark at 25°C for 7 days. For plasmid construction, preparation, and propagation, Escherichia coli DH5α (Tiangen, Beijing, China) was used and grown in LB medium, supplemented with ampicillin (100 μg/ml) or kanamycin (50 μg/ml) at 37°C for 24 h. *Agrobacterium tumefaciens* strain GV3101 (Tiangen, Beijing, China) was grown on minimal medium (MM) at 28°C for 2 days (Lei et al., 2017). An induction medium (IM) with 100 μg/ml of hygromycin (Hyg, Roche, USA) was used to co-cultivate *A. tumefaciens* and protoplasts of *P. ostreatus*. Transformants of *P. ostreatus* were grown on complete medium (CM) for cultivation at 25°C under darkness for 10 days (Lei et al., 2017). Then, for testing response to *T. putrescentiae* grazing, the WT strain and its transformants were cultivated on PDA within a six-well plate for 5 days or wheat grains with a 25 ml conical flask for 10 days at 25°C (Hou et al., 2022).

### 2.2. Mite stock

The populations of *T. putrescentiae* were maintained at the Institute of Vegetable Research, Jiangsu Academy of Agricultural Sciences, Nanjing, Jiangsu, China. *T. putrescentiae* was reared in a round plastic transparent box (a diameter of 7 cm) containing the vegetative mycelia of *Lentinula edodes* at 26 ± 1°C and 80 ± 5% relative humidity (RH) (Hou et al., 2022). Two months before the experiment, populations to transferred to wheat grain straws of the WT strain and its transformants at 28°C to assess host suitability.

### 2.3. *Polec2* gene cloning and transplantation

After 7 days of growth on PDA at 25°C, *P. ostreatus* mycelia were harvested and frozen in liquid nitrogen, and then stored at −80°C. Total RNA was extracted from 0.1 g of each sample using an SV Total RNA isolation kit (Promega, WI, United States), then reverse transcribed to cDNA with a First-stand cDNA Synthesis Kit (Takara, Beijing, China) as described previously (Hou et al., 2022). The *Polec2* sequence was amplified by an I-5TM 2X high-fidelity master mix polymerase (Tsingke, Beijing, China) using the primers PoLec2-1F (5′-ATGCATGACATTAGTACTTAT-3′) and PoLec2-1R (5′- CTATGCGAG-AGGGATGACTAC-3′) designed from the transcriptome data (accession number: PRJNA665192) of the predicted lectin 2 of *P. ostreatus*. The PCR conditions were as follows: 98°C for 5 min, followed by 30 cycles of denaturation at 98°C for 15 s, annealing at 55°C for 10 s, and elongation at 72°C for 40 s, and a final elongation step at 72°C for 10 min. The PCR product was purified and cloned into the pMD19-T vector and then sequenced (Tsingke, Nanjing, China).

The *Polec2* cDNA was purified by agarose gel electrophoresis and ligated to the pCAMBIA1303 vector (Abcam, Shanghai, China). Protoplasts of *P. ostreatus* were prepared as described (Nurziya et al., 2019). The transformation method of *P. ostreatus* was described performed according to Shi et al. (2012). The stability of transformants was evaluated by the relative expression of *Polec2* using quantitative real-time PCR (qRT-PCR) analysis after they were grown on CM agar without hygromycin for five rounds of cultivation. The qRT-PCR primer pairs of *Polec2* were shown in Supplementary Table S1. The β-actin gene was used as an internal standard.

### 2.4. Phylogenetic analysis

One hundred thirty seven lectin sequences from 22 mushroom species were obtained from the MycoLec module of UniLectin^1^ and the NCBI database^2^ (Supplementary Table S2), and clustered using the MAFFT tool with a global matching algorithm (the maximum number of iterative refinements was 1,000) (Yamada et al., 2016). The best-fit algorithm was tested using the Modomatic method integrated into iqTree as WAG+R5 (Yamada et al., 2016), which was then used to calculate the genetic evolution tree in iqTree using the maximum likelihood (ML) method with a Bootstrap value of 5,000 times (Whelan et al., 2015; Minh et al., 2020). Tree files were generated and annotated using the iToL online tool (Letunic and Bork, 2021).

### 2.5. Antioxidant activity assay

Fresh mycelia of the WT strain and its transformants were collected after cultivation on 100 ml PDB at 25°C for 7 days. Using commercially available detection kits (Webiolotech, Nanjing, China), the total antioxidant catalase (CAT) activity, peroxidase (POD) activity, superoxide dismutase (SOD) activity, H_2_O_2_ content, and malondialdehyde (MDA) content were measured. These tests were performed using 1.0 g of fresh mycelia of each transformant and were repeated four times. The WT strain was used as a control.

### 2.6. Analysis of hormone content changes

Mycelial samples fresh weight 1.0 g *per capita* were crushed in liquid nitrogen and stored at −80°C in glass tubes. The production of salicylic acid (SA), methyl salicylate (MeSA), jasmonic acid (JA), 12-oxo-phytodienoic acid (OPDA), jasmonic acid-isoleucine (JA-Ile), jasmonic acid methyl ester (MeJA), indole-3-acetic acid (IAA), 3-indole butyric acid (IBA), abscisic acid (ABA), indole-propionic acid (IPA), and isopentenyladenine (IP) were analyzed using a high-performance liquid chromatography–tandem mass spectrometry (HPLC-MS/MS) method with a QTRAP 6500 triple quadrupole mass spectrometer of AB Sciex (Framingham, MA, United States). The pretreatment and detection procedure of samples were performed as described previously (Webiolotech, Nanjing, China) (Li et al., 2022).

### 2.7. Expression analysis of Key genes in the immune defense system by qRT-PCR

The expression of 14 key genes in the defensive signaling pathway was analyzed in the transgenic strains using an one-step qRT-PCR kit (Takara, Tokyo, Japan) with the Light-cycling 480 Real-Time System (Roche, Switzerland) (Supplementary Table S2). Total RNA of transgenic strains and subjected to Primer Quest™ Tool^3^ for qRT-PCR were designed from the *P. ostreatus* CCMSSC0389, PC15 and PC9 reference genome (PRJNA476433, PRJNA670761 and PRJNA81933) and the transcriptome data in Primer 6.0 software (Supplementary Table S2). The PCR condition used 95°C for 2 min, followed by a cycling program of 95°C for 10 s, 58°C for 30 s, and 72°C for 30 s, and then the productions were heat denatured over a 35°C temperature gradient at 2.2°C/s from 60°C to 95°C (Li et al., 2022). The β-actin gene was used as an internal standard. A standard curve was constructed for every set of primers using a cDNA sample diluted using a 10-fold gradient (Supplementary Table S1). The gene expression levels in the WT strain mycelia were the control. Three biological replicates were analyzed per sample. Relative gene expression levels were computed according to the 2^-▵▵CT^ method described by Winer et al. (1999).

### 2.8. Assay for *Polec2*-overexpressing strains resistance against *Tyrophagus putrescentiae*

*Polec2*-overexpressing strains resistance against *T. putrescentiae* was assessed as development, survivor ship and host fitness parameters of *T. putrescentiae* reared on the fresh mycelia using at least six replicates of each strain as previously described (Hubert et al., 2013; Hou et al., 2022). Before the start of these tests, *T. putrescentiae* was exposed to the WT strain and transgenic strains for five generations.

### 2.9. Statistical analysis

The statistical analysis of experimental data was performed using IBM SPSS 26.0 (IBM Corp., NY, United States). The data were examined for homogeneity of variance before analysis. Differences in the transgenic strains’ hormone level, gene expression, antioxidant activity, and the biological parameters of *T. putrescentiae* were evaluated using a one-way ANOVA test, followed by a Waller-Duncan test (*p* = 0.05).

## 3. Results

### 3.1. Sequence analysis, gene structure, and phylogenetic analysis of *Polec2*

**Figure 1:**
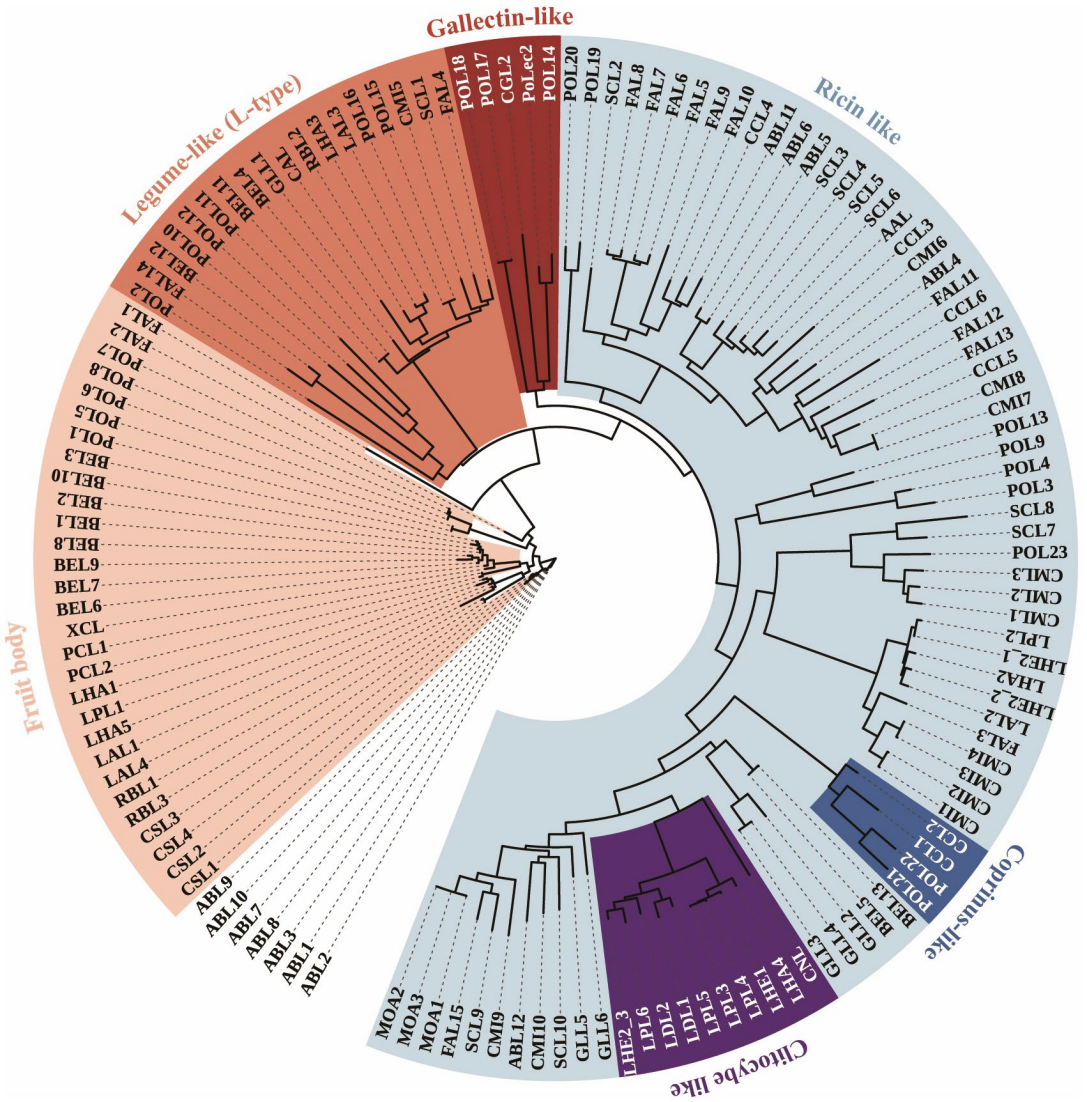
Phylogenetic tree of 137 lectin proteins from 22 species in Agaricomycetes. Unrooted phylogenetic tree of lectins protein sequences from *Pleurotus ostreatus* (POL1-23 and PoLec2), *P. cornucopiae* (PCL1 and PCL2), *Agaricus bisporus* (ABL1-8), *Auriculariopsis ampla* (AAL), *Clitocybe nebularis* (CNL), *Coprinellus micaceus* (CML1-10), *C. cinerea* (CCL1-6), *Cubamyces menziesii* (CML1-3), *Cyathus striatus* (CSL1-4), *Flammula alnicola* (FAL1-15), *Schizophyllum commune* (SCL1-10), *Boletus edulis* (BEL1-13), *Xerocomellus chrysenteron* (XCL), *Cantharellus anzutake* (CAL), *Ganoderma leucocontextum* (GLL1-6), *Lactarius akahatsu* (LAL1-4), *L. deliciosus* (LDL1 and LDL2), *L. hatsudake* (LHA1-5), *L. hengduanensis* (LHE1 and LHE2_1- LHE2_3), *L. pseudohatsudake* (LPL1-6), *Macrolepiota procera* (MOA1 and MOA2), and *Russula brevipes* (RBL1-3). The outer ring indicates the classes of lectins, including Galectins (shaded in red), Coprinus-like (shaded in blue), Ricin-like (shaded in cyan), Clitocybe-like (shaded in purple), Legume-like (shaded in orange), and Fungal fruit body (shaded in pink) lectins. The tree is displayed using the iTOL web server.

A total putative full-length cDNA of *Polec2* was cloned (GenBank accession number: OP751397), encoding a putative protein of 431. *Polec2* has a molecular weight of 45.77 KDa and a predicted pI of 8.39. Based on the phylogenetic relationship, 137 lectins from 22 different species of mushroom-forming fungi, including 12 *Agaricales*, two *Boletales*, one *Cantharellales*, one *Polyporales*, and six *Russulales*, were clustered into 8 different groups in the ML tree (Figure 1). Agaricales display the largest variety, spanning over all of 6 classes of lectins. Strong variations in lectin catalogs from *P. ostreatus* are observed depending on the fungal lineage, with 24 lectins were clustered into 5 different classes. Some lectin classes such as Coprinus-like (CCL-like) β-trefoil-type lectins were frequently identified within two *Pleurotus* species. *Polec2* was classified to the galectin-like class with β-sandwich-fold.

Six groups of these proteins were reported to be involved in the fungal defense of the fungal fruit body, including galectins, Coprinus-like, ricin-like, Clitocybe-like, and fungal fruit body lectins (Varrot et al., 2013; Tayyrov et al., 2018; Bleuler-Martinez et al., 2022; Figure 1). For example, lectins with entomotoxic activity, CNL from *Clitocybe nebularis* is Clitocybe-like lectin family, MPL from *Macrolepiota procera* are ricin-like lectin family, CCL2 from *Coprinopsis cinerea* is galectins lectin family, and XCL from *Xerocomellus chrysenteron* is fungal fruit body lectin family (Wohlschlager et al., 2014; Bleuler-Martinez et al., 2022). Furthermore, the ricin-like lectin family is the major group (46.7%) of 137 proteins, those mostly contain β-trefoil fold structures from *Schizophyllum commune*, *Coprinellus micaceus*, *Flammula alnicola*, etc., in more closely related branches, which are likely to be of value in providing potential insecticidal activity. Structurally, POL21 and POL22 from *P. ostreatus* had higher homologies to CCL2, having potential for entomotoxic activity that remains to be verified.

### 3.2. Regulation of antioxidant enzyme activities

**Figure 2:**
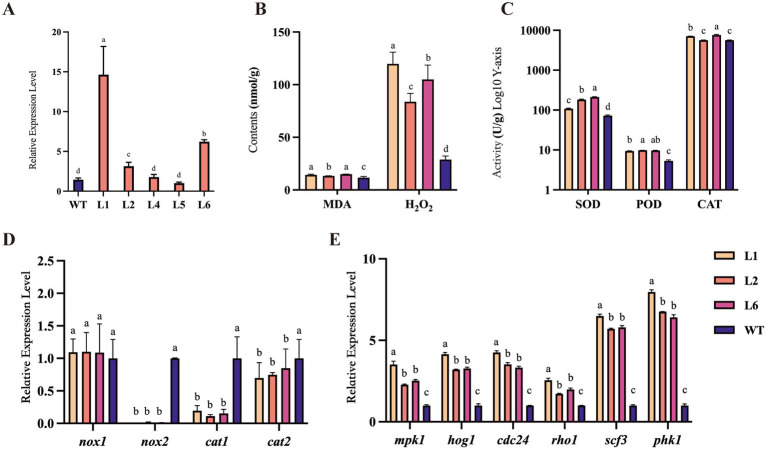
The changes of antioxidant enzyme activities in *Polec2* overexpressing strains. **(A)** Relative expressions of *Polec2* in the six transformants (L1 to L6) after grown on complete medium without hygromycin for five rounds of cultivation. **(B)** Change in malondialdehyde (MDA) and hydrogen peroxide (H_2_O_2_) contents. **(C)** Change in superoxide dismutase (SOD), peroxidase (POD), and catalase (CAT) activities. **(D)** Expression analysis of genes in the reactive oxygen species (ROS) metabolism, including the NADPH oxidases catalytic subunits genes *nox1* and *nox2*, and the catalase genes *cat1* and *cat2*. **(E)** Expression analysis of genes in the mitogen-activated protein kinases (MAPK) signaling pathway, including MAPKs genes *hog1* and *mpk1*, serine/threonine-protein kinases *Pkh1*, and guanine nucleotide exchange factors *pho1*, *scf3*, and *cdc24*. Letters indicate significant differences from the WT strain (*p* < 0.05, Waller-Duncan test).

Subsequently, we obtained five stable transformants from six overexpressing strains (named L1 to L6) of *Polec2* in *P. ostreatus* by the *Agrobacterium*-mediated transformation. According to the qRT-PCR analysis, the transformants all showed significantly elevated *Polec2* expression relative to the WT strain, with L1, L2 and L6 being most upregulated (Figure 2A). These three transformants were further used to evaluate their bioactivity, including hormone level, gene expression, and antioxidant activity.

ROS is important in various biological functions, including cell survival, cell growth, proliferation, differentiation, signaling, immune response, and other physiological responses (Zuo et al., 2015). Three key antioxidant enzymes involved in the biosynthesis and degradation of ROS were measured to investigate the antioxidative effects of *Polec2*. For L1 and L6 strains, SOD, POD, and CAT activities of fresh mycelium significantly increased than those in WT strain (*F*_3,15_ = 178.2–1116.1, *p* < 0.001) (Figure 2C). An increase in the content of H_2_O_2_ (*F*_3,15_ = 65.9, *p* < 0.001) was also observed (Figure 2B). MDA is one of the most significant byproducts of membrane lipid peroxidation, and its activity was strongly activated in the transformants (*F*_3,15_ = 18.7, *p* < 0.01), which subtly indicated the membrane systems’ greater stress resistance (Figure 2C). In addition, we investigated the expression levels of the key genes involved in the ROS metabolism, including the NADPH oxidases catalytic subunits coding genes, *nox1* and *nox2*, and the catalase genes *cat1* and *cat2*. Compared to unaffected *nox1* expression, the expression of *nox2* was abolished in three transformants, accompanied by differential downregulation of *cat1* and *cat2* (Figure 2D). Compared with L1 and L6 strains, CAT activities of L2 strain showed no difference with WT (Figure 2C).

Furthermore, *Polec2* overexpression activated gene expression in the mitogen-activated protein kinases (MAPK) pathway (Figure 2E). MAPK kinases HOG1 and MPK1, and guanine nucleotide exchange factors *pho1*, *scf3*, and *cdc24* were all significantly increased in the transformant L1, ranging from 2.54 to 8.0-fold, whereas *mpk1, hog1*, *pho1*, and *scf3* were not significantly changed in the transformant L2 and L6 (Figure 2E). The relative expression of serine/threonine-protein kinases PKH1 was all significantly increased in the three transformants. ROS/MAPKs signaling pathway plays an important role in direct anti-fungivory defenses of the oyster mushroom.

### 3.3. Activation of salicylic acid- and jasmonic acid-signaling pathways in *Polec2* overexpressing strains

**Figure 3:**
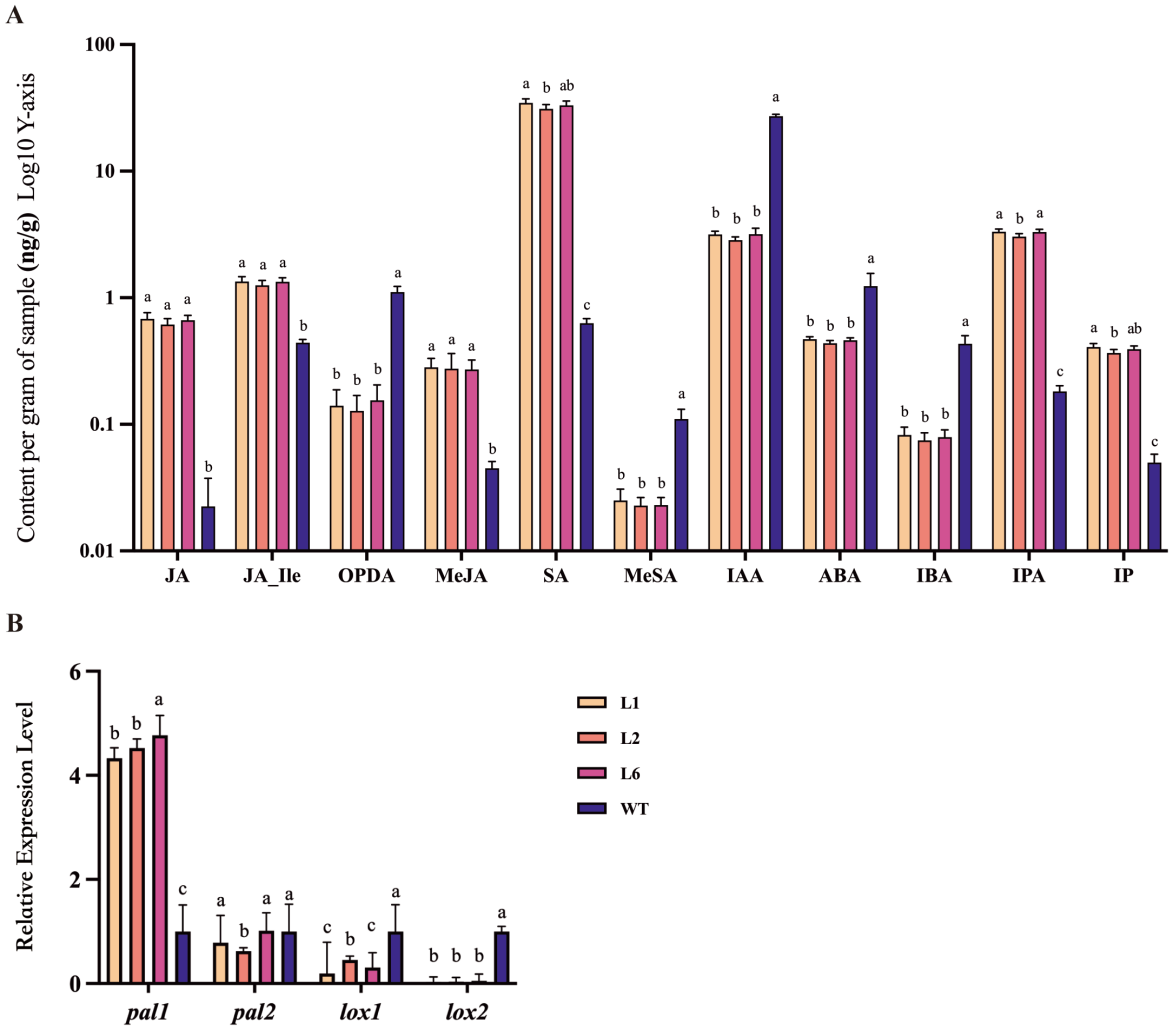
The changes of hormone levels in *Polec2* overexpressing strains (L1, L2 and L6). **(A)** Change in different hormone levels (using a log scale), including the production of salicylic acid (SA), methyl salicylate (MeSA), jasmonic acid (JA), 12-oxo-phytodienoic acid (OPDA), jasmonic acid-isoleucine (JA-Ile), jasmonic acid methyl ester (MeJA), indole-3-acetic acid (IAA), 3-indole butyric acid (IBA), abscisic acid (ABA), in-dolepropionic acid (IPA), and isopentenyladenine (IP). **(B)** Expression analysis of genes in the SA- and JA-signaling pathways, including two phenylalanine ammonia-lyases *pal1* and *pal2* for SA biosynthesis and two Lipoxygenases *lox1* and *lox2* for JA biosynthesis. Letters indicate significant differences from the WT strain (p < 0.05, Waller-Duncan test).

To examine the effect of *Polec2* on *P. ostreatus* defense against *T. putrescentiae*, we measured the levels of JA, JA-Ile, OPDA, MeJA, SA, MeSA, IAA, ABA, IBA, IPA, and IP in *Polec2* overexpressing strains. Compared with the WT strain, the levels of SA, JA, JA-Ile, MeJA, IP and IPA were significantly higher (*F*_3,15_ = 16.705–252.359, *p* < 0.0001), whereas the levels of MeSA, OPDA, IAA, IBA, and ABA were significantly lower (*F*_3,15_ = 23.828–2053.547, *p* < 0.0001, respectively; Figure 3A). Moreover, the level of SA rose over 50-fold. The expression of the SA- and JA-signaling pathways proteins, and phenylalanine ammonia-lyase 1 (PAL1) were significantly up-regulated (4.33-fold, 12.47-fold, and 1.77-fold, respectively), whereas PAL2 not increased in the transformants. Lipoxygenase 1 and 2 (LOX1 and LOX2) were not induced (Figure 3B). In contrast, *Polec2* overexpression strongly strengthens SA- and JA-dependent defense of *P. ostreatus*.

### 3.4. *Polec2*-overexpressing strains resistance analysis

**Figure 4:**
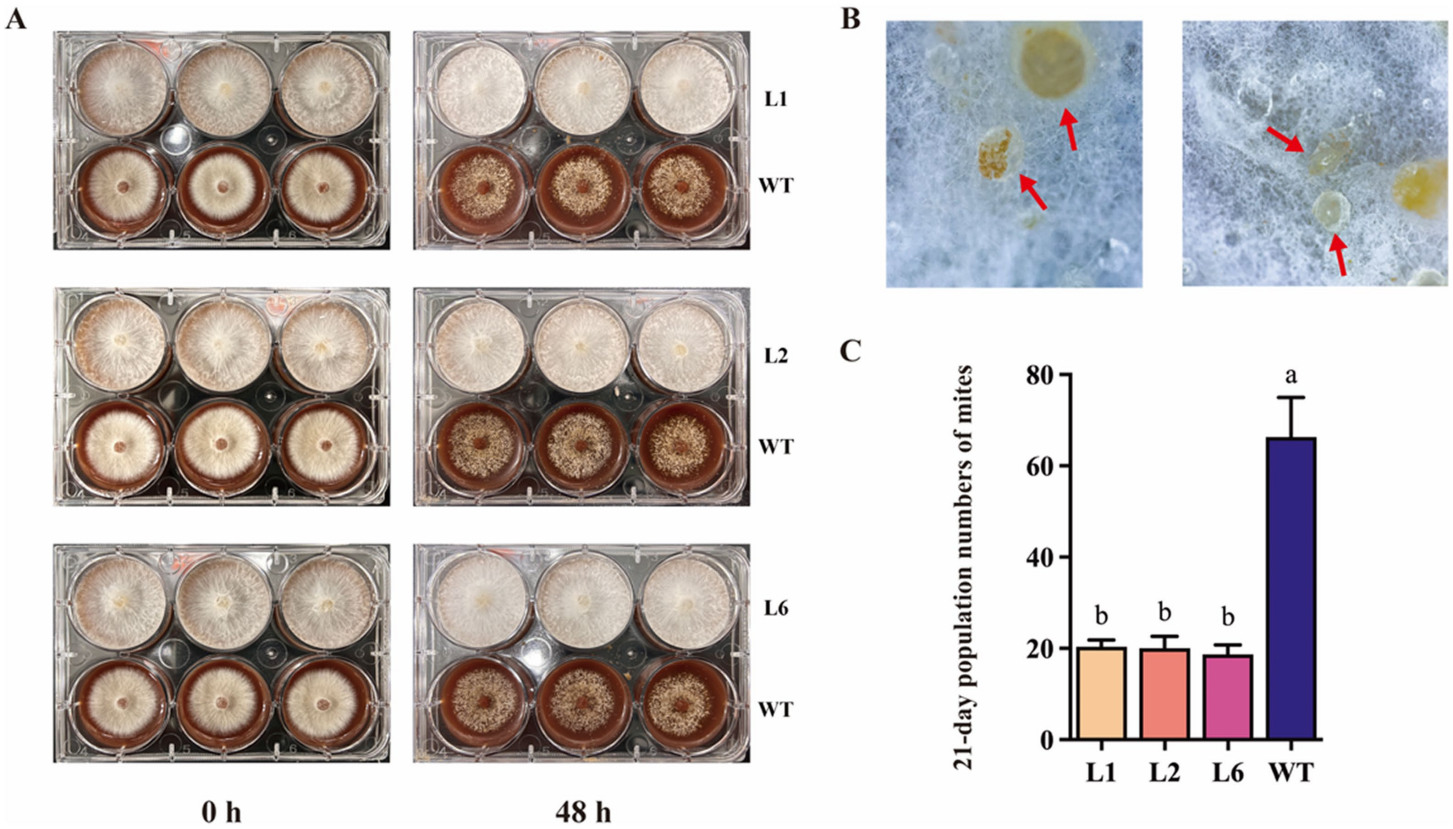
*Polec2* overexpressing strains resistance to *T. putrescentiae*. **(A)** Infestation tests in *Polec2* overexpressing strains (framed into blue square). **(B)** *T. putrescentiae* (red arrow) was buried in the mycelium of *Polec2* overexpressing strains. **(C)** The 21-day population numbers of *T. putrescentiae* feeding on *Polec2* overexpressing strains (L1, L2 and L6). Letters indicate significant differences from the WT strain (*p* < 0.05, Waller-Duncan test).

To investigate how *Polec2* influences *T. putrescentiae* feeding, growth, development and population increase, infestation tests were performed on L1, L2 and L6 transformants. The mycelium of the WT strain was almost depleted after 48 h of *T. putrescentiae* feeding, but the mycelium of the transformants L1, L2 and L6 had only small holes (Figure 4A). By careful examination, we could see that the mites are buried and are feeding on the area where the mycelium nuclei have twisted into clusters. The mites could be seen feeding on the mycelium that had knotted into clusters but became entangled, then died (Figure 4B). On the contrary, the white mycelium of WT strain was significantly reduced and sparse after 48 h of mite feeding (Figure 4A). However, we found that the mites could complete the life history in the transformants, and the total developmental stages were not significantly different from the WT strain (Table 1). Instead of growth, the hatchability and survival of *T. putrescentiae* were significantly reduced (by 12–22%) (Table 1). The rate of population increase of *T. putrescentiae* was estimated and incubated in 25 ml culture flasks with 0.25 g grains of tested strains for 21 d at 25°C and 85% RH. The population rate of increase was strongly negative for the transformants, about 25% of that for the WT strain (Figure 4C).

**Table 1:** The developmental durations (days) for *T. putrescentia*e reared on *Polec2* overexpressing strains at 25°C and 85% RH.

Strains	Egg	Larva	Deutonymph	Tritonymph	Total	Hatchability	Mortality
L1	2.51 ± 0.77	2.5 ± 0.68a	3.11 ± 1.4	2.26 ± 0.79ab	10.54 ± 0.94	82.43 ± 3.82b	82.39 ± 3.08b
L2	3.12 ± 3.3	1.91 ± 0.89ab	3.31 ± 3.56	1.1 ± 2.41b	9.99 ± 1.54	85.33 ± 6.91b	79.41 ± 2.79b
L6	2.1 ± 0.58	1.77 ± 0.53ab	2.28 ± 0.96	3.5 ± 1.25a	10.03 ± 1.47	85.73 ± 3.08b	76.31 ± 2.16b
WT	2.28 ± 0.5	1.54 ± 1.27b	3.32 ± 0.77	2.36 ± 1.35ab	9.96 ± 2.1	98.54 ± 1.84a	98.43 ± 2.61a

Values are presented as mean ± SD.

Means within a column followed by the same letter are significantly different (Waller-Duncan test: *p* < 0.05).

## 4. Discussion

Fungi are eukaryotic organisms that absorb nutrients and can be found in any ecological niche. Fungi have been studied in the laboratory as model systems in every aspect of the biological sciences due to their simple and rapid life cycles, yet complex genetics, metabolism, and morphology (Biedermann and Vega, 2020). Fungi frequently use predation, parasitism, and antagonism to suppress their competitors, including agricultural pests (Ambethgar, 2009). Most fungi used to control agricultural pests are Hyphomycetes, capable of infecting and killing insects and are thus referred to entomopathogenic fungi (Shamshad, 2010). Conversely, some pests can destroy edible fungal production, which is already a significant economic crop worldwide (Qu et al., 2015; Panevska et al., 2019). To avoid harmful chemicals, further research is needed on integrated pest management in mushrooms, biological control, and in-depth insight into edible fungal defense mechanisms.

As a part of their chemical defense, edible fungi have been shown to produce terpenoids, glycans, aegerolysins, lectins, and other substances (Kunzler, 2015; Lebreton et al., 2021; Li et al., 2022). Several aegerolysins have been found to have insecticidal activity in *Pleurotus* mushrooms, including ostreolysin A (OlyA) and pleurotolysin A (PlyA), pleurotolysin B (PlyB), and erylysin A (EryA) with the membrane-attack-complex/ perforin domain, which can bind to insect cells and artificial lipid membranes and toxicity to western corn root-worm larvae and adults and Colorado potato beetle larvae (Wohlschlager et al., 2014; Panevska et al., 2019). More than 13 types of lectins have been found in the genome of *P. ostreatus*, including the AAL-like lectin, oyster lectin, fungal fruit body lectin, and Ricin B lectin (Perduca et al., 2020). Here, our study showed strong variations in lectin catalogs from *P. ostreatus*, and found new classes including CCL-like β-trefoil-type and galectin-like class with β-sandwich-fold lectins. Significantly, CCL-like β-trefoil-type lectins in other fungi species have previously been reported to have resistance against predators and parasites (Bleuler-Martinez et al., 2017; Vajravijayan et al., 2020; Lebreton et al., 2021).

In the present study, *Polec2* was proven to activate ROS/MAPK signaling and reinforce SA- and JA-dependent defense of *P. ostreatus* against *T. putrescentiae*. After overexpression of *Polec2* (more than 5-fold), two of the signaling events were the activations of ROS and MAPKs. As a result of an increase in generation of ROS, ROS-scavenging proteins such as protein SOD, CAT and POD were induced upon cell damage. Furthermore, MDA content are essential biological parameters for evaluating the degree of oxidative cell damage (Mittler et al., 2011; Hernández-Oñate et al., 2012). These results indicate that overexpression of *Polec2* could significantly induce the accumulation of MDA content and decrease electrolyte permeability. NADPH oxidases (Noxs) have a variety of functions in plants and animals, including response to wounding, predators, and programed cell death (PCD), where ROS produced by Noxs forms a gradient of H_2_O_2_, indicating that it serves as propagation signal (Mittler et al., 2011). The advantage of ROS includes the capacity of the cell to rapidly produce and scavenge different forms of ROS in a simultaneous manner, enabling rapid and dynamic changes in ROS levels. Different organisms generate ROS at different levels and could leak or actively transport ROS such as H_2_O_2_ into their environment, it is possible that another advantage of ROS as signaling molecules in early stages of evolution was the sensing and/or communication between different organisms (Mittler et al., 2011). In fungi, wounding causes two of three Noxs (*nox1*, *nox2*, and *noxR*) dependent ROS production in the fungus *Trichoderma atroviride*, and *noxR* protein regulates Nox1 according to gene-replacement experiments (Hernández-Oñate et al., 2012; Medina-Castellanos et al., 2014). Fungi contain from one to three Nox genes, depending on the species (Aguirre et al., 2005). We discovered that one of two Nox genes, Nox1, had the potential ability to regulate ROS production in *P. ostreatus*, but this conclusion need further investigation. These findings suggested that, as in plants and animals, changes in intracellular redox status may act as signal molecules in a conserved defense-response mechanism in fungi (Aguirre and Lambeth, 2010). Activation of MAPKs signaling confirms its important role in the oyster mushroom defense, which could involve signaling multiple defense responses, including the biosynthesis/signaling of defense hormones, ROS generation, defense gene activation, and antibiotic substance production (Li et al., 2022). And then SA and JA biosynthesis and metabolism were found to both strongly increase in *Polec2* transgenic strains when compared to the WT strain. SA and JA have been discovered to be crucial components of *P. ostreatus*’ defense systems against *T. putrescentiae* attack in earlier investigations (Li et al., 2022). In particular, endogenous and exogenous JA can influence the expression of genes involved in the biosynthesis of terpenoids and steroids to produce the metabolism of anti-mite substances (Li et al., 2022). Overall, our results show that *Polec2* actives the ROS, MAPK, SA, and JA signaling pathways to defend against *T. putrescentiae* infestation. In this study, we discovered that rearing *T. putrescentiae* on *Polec2* transgenic strains significantly reduced hatchability, survival, and population increase, indicating stronger resistance to *T. putrescentiae*.

According to these observations, *Polec2* expression could be beneficial in efforts to control *T. putrescentiae* without using other inputs or causing environmental damage. This study also presents a method for elucidating resistance mechanisms in edible fungi, providing a resistance-conferring gene for breeding mite-resistant mushroom varieties in pest control. Interestingly, *P. ostreatus* appeared to produce some sticky particles that limited the action of *T. putrescentiae* and caused its death, though the underlying mechanism is unknown and should be investigated in future work.

## Data availability statement

The original contributions presented in the study are included in the article/Supplementary material, further inquiries can be directed to the corresponding authors.

## Author contributions

CL and SQ: conceptualization. HL: methodology. JL and XL: software. JL: writing original draft preparation. SQ: writing review and editing. LM: project administration. SQ and LM: funding acquisition. All authors have read and agreed to the published version of the manuscript.

## Funding

This research was funded by the National Natural Science Foundation of China (32001911), the China Agriculture Research System of MOF and MARA (CARS-20), and Biological Quality Engineering Project (503190106).

## Conflict of interest

The authors declare that the research was conducted in the absence of any commercial or financial relationships that could be construed as a potential conflict of interest.

## Publisher’s note

All claims expressed in this article are solely those of the authors and do not necessarily represent those of their affiliated organizations, or those of the publisher, the editors and the reviewers. Any product that may be evaluated in this article, or claim that may be made by its manufacturer, is not guaranteed or endorsed by the publisher.

## Supplementary material

The Supplementary material for this article can be found online at: https://www.frontiersin.org/articles/10.3389/fmicb.2023.1191500/full#supplementary-material

## Footnotes

^1^ https://www.unilectin.eu/mycolec/

^2^ http://www.ncbi.nlm.nih.gov

^3^ https://sg.idtdna.com/pages/tools/primerquest
