# Supplementary material for: A lectin gene is involved in the defense of Pleurotus ostreatus against the mite predator Tyrophagus putrescentiae
Source: Front Microbiol. 2023 Apr 27;14:1191500. doi: 10.3389/fmicb.2023.1191500 (PMC10174108; doi:10.3389/fmicb.2023.1191500)
Supplement: Supplementary file 1 [file Table_1.docx]

**Table S1 Primers used in the qRT-PCR reactions of gene expressions in this study**

| **NO** | **Gene** | **Primer name** | **Primer sequence (5' to 3')** | **Length(bp)** | **Standard curve** |
| --- | --- | --- | --- | --- | --- |
| 1 | *β*-*Actin* | ACTIN-F | AGTCGGTGCCTTGGTTAT | 18 | 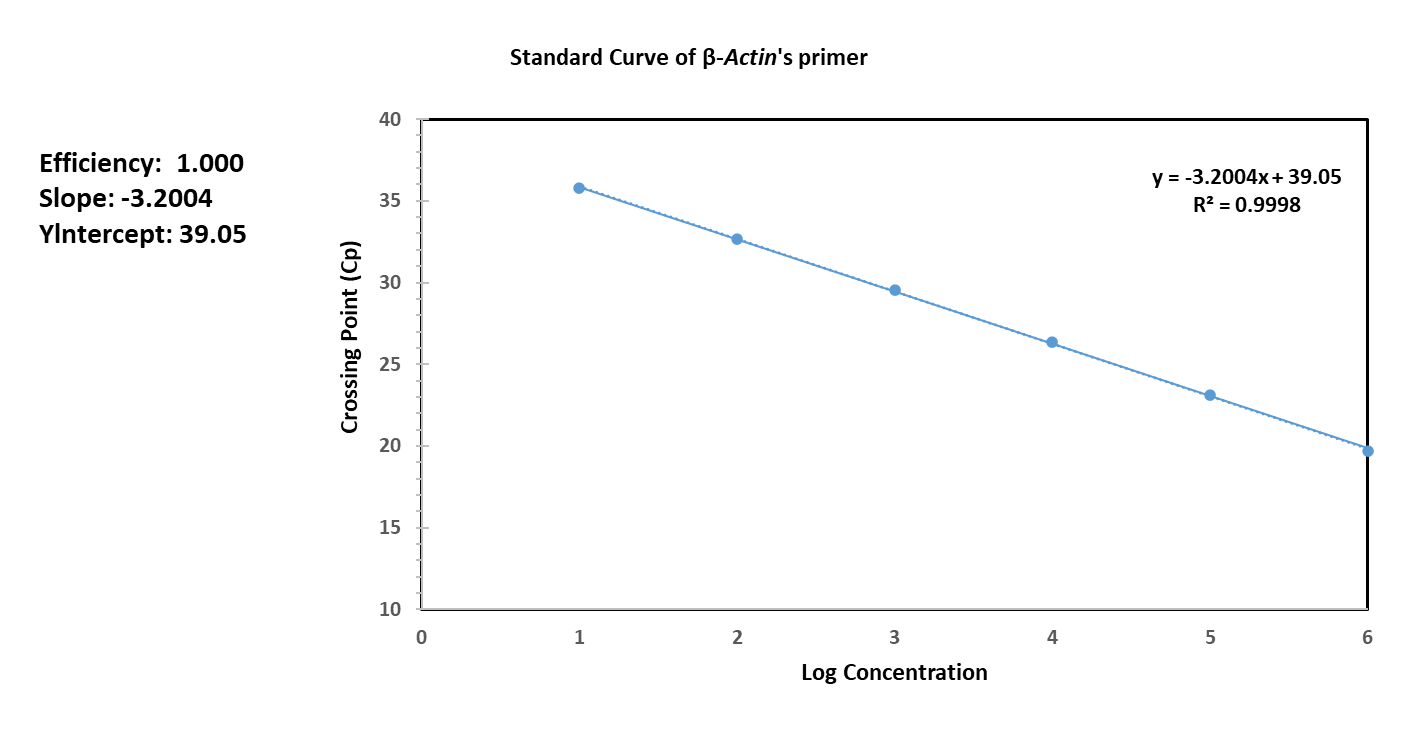 |
|  |  | ACTIN-R | ATACCGACCATCACACCT | 18 |  |
| 2 | *PoLec2* | LEC2-F | GAGCACCGACACCTTCATCA | 20 | 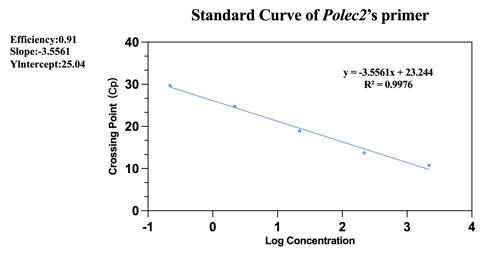 |
|  |  | LEC2-R | AATCCCCACTTGCTGTCGTC | 20 |  |
| 3 | *Nox1* | NOX1-F | CTTCCCAGCCGATGAGAATATC | 22 | 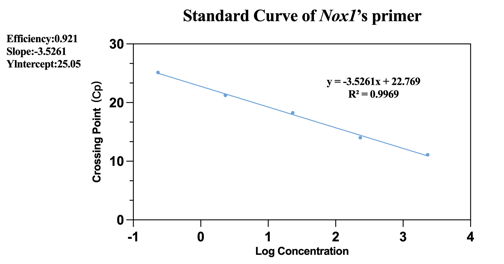 |
|  |  | NOX2-R | AGTTGACGTAGTGAGCTGTTG | 21 |  |
| 4 | *Nox2* | NOX2-F | GTTCAATCTCTGGATGATTAACG | 23 | 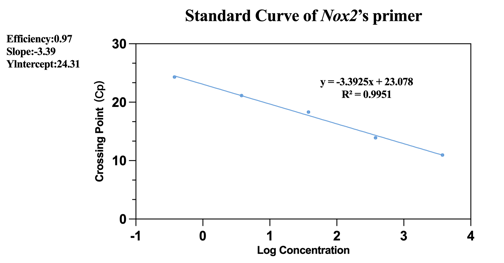 |
|  |  | NOX2-R | TTAGAAGTTCTCCTTTCCGAAG | 22 |  |
| 5 | *Cat1* | CAT1-F | GACCCCACCGATAGTTCCAC | 20 | 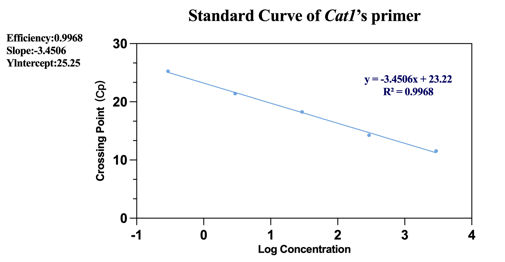 |
|  |  | CAT1-R | TCTCACCAATGCTCAGGGTC | 20 |  |
| 6 | *Cat2* | CAT2-F | CAGGATCTTTCGGACCGCAT | 20 | 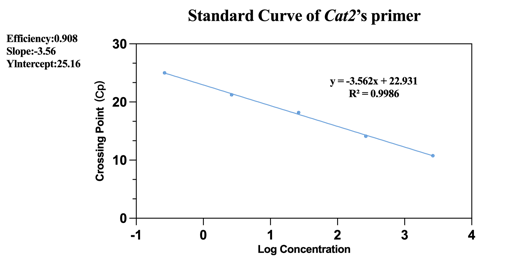 |
|  |  | CAT2-R | GGTGGACTTGAAGGCACCTA | 20 |  |
| 7 | *Rho1* | RHO1-F | CCCAACGGTCTTCGAGAACT | 20 | 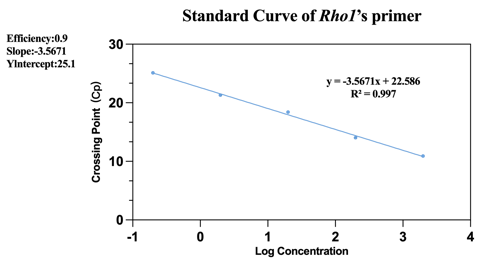 |
|  |  | RHO1-R | CGGCCTGAGGCGATCATATT | 20 |  |
| 8 | *Hog1* | HOG1-F | GAGTAGTCCACCGCGATCTG | 20 | 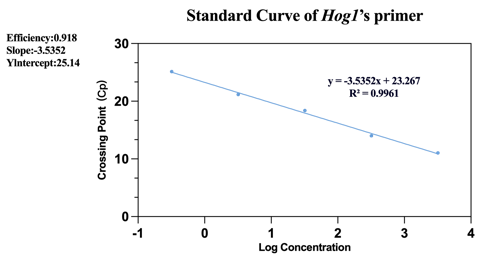 |
|  |  | HOG1-R | CGTCATTTGGGGGTCTTGGA | 20 |  |
| 9 | *Cdc24* | CDC24-F | AGTCGCTGCGAAACGCATTA | 20 | 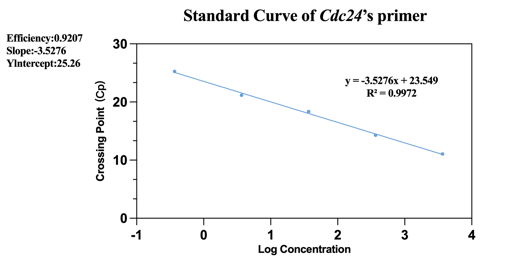 |
|  |  | CDC24-R | TGGTGCCCTTTCCAATCTCC | 20 |  |
| 10 | *Mpk1* | MPK1-F | CCTTCTGTCCCGAATGCTATG | 20 | 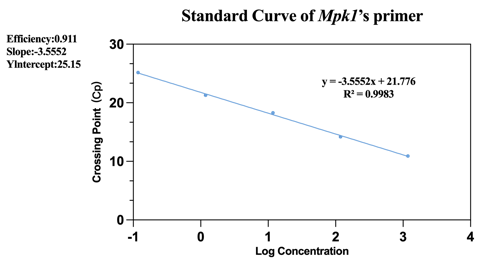 |
|  |  | MPK1-R | TCGTGCCAAACTTGGAGATAC | 20 |  |
| 11 | *Phk1* | PHK1-F | CGGATACAACACCGTTCCTATAC | 21 | 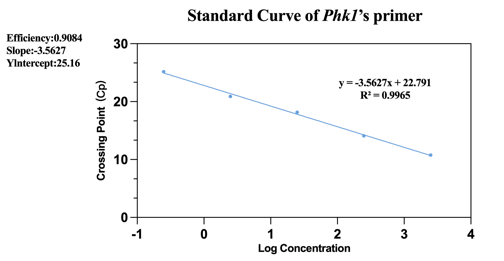 |
|  |  | PHK1-R | CGAAACACCCGCTACATTCT | 21 |  |
| 12 | *Scf3* | SCF3-F | TCCAAGTTCAGCACCCTAAAG | 23 | 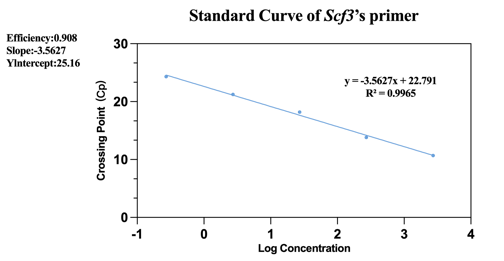 |
|  |  | SCF3-R | GAGGAAGTTGAGTCTACGGATAAG | 26 |  |
| 13 | *Lox1* | LOX1-F | ACCAAACAAACCATCGGCAC | 20 | 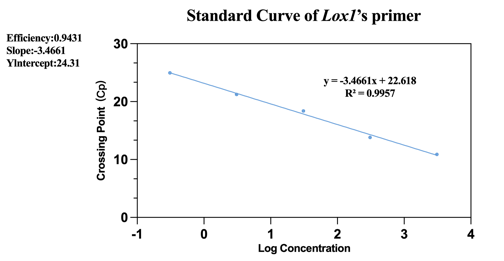 |
|  |  | LOX1-R | GTTTGGCATTGAGGGGGAGA | 20 |  |
| 14 | *Lox2* | LOX2-F | CAACTCAAAAACCTCGGGCG | 20 | 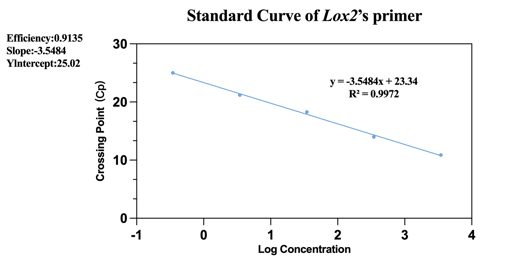 |
|  |  | LOX2-R | GAGTTCGGTGGTGGGTCTTT | 20 |  |
| 15 | *Pal1* | PAL1-F | CTGTCGCTTCTAATCTCCTCATAC | 24 | 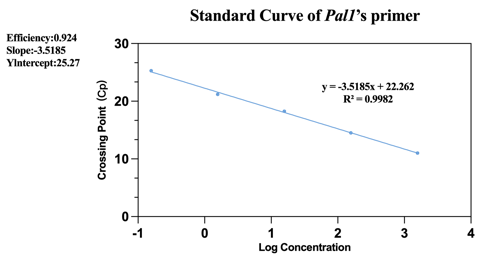 |
|  |  | PAL1-R | TAATTTCTTCGACGCCCTTCA | 21 |  |
| 16 | *Pal2* | PAL2-F | CGCACAGTATCTCGCCTATATC | 22 | 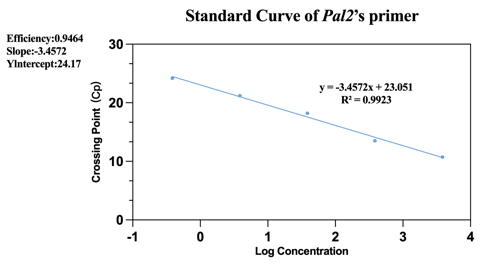 |
|  |  | PAL2-R | GTACTTGGGTGAGCAGAGTAAG | 22 |  |
