# Supplementary material for: A lectin gene is involved in the defense of Pleurotus ostreatus against the mite predator Tyrophagus putrescentiae
Source: Front Microbiol. 2023 Apr 27;14:1191500. doi: 10.3389/fmicb.2023.1191500 (PMC10174108; doi:10.3389/fmicb.2023.1191500)
Supplement: Supplementary file 2 [file Table_2.docx]

**Table S2** **The sequences of all Lectin proteins in this study**

| **No.** | **Gene No.** | **Species** | **ORF sequences** |
| --- | --- | --- | --- |
| 1 | ABL1 | *Agaricus bisporus* | MSYTVTVRVYQTNPNTFFRRVEETVWKYANGGTWDEAKGEYVLTMGGSGTSGSLRFISDTGERFVATFGVHNYKRWCDIVTDLTDEQTALVINQEYYGVPERDQARERQLESYNVSNAKGRNFAVKYTITGGNHLKANLIIG |
| 2 | ABL2 | *A. bisporus* | MSYTITIRVYQTDPNAFFRRVEQTCWKYANGCTWDEAKGEYVLTMGGSGTSGSLRFVSDTGERFVATFGVHNWKRWCDIVTNLTDEQTALVINQEYYGVPYRDAARERQLTSYSVSDREGRKYTINYTVTDGNNLKANLIIG |
| 3 | ABL3 | *A. bisporus* | MTYTISIRVYQTTPKGFFRPVERTNWKYANGGTWDEVRGEYVLTMGGSGTSGSLRFVSSDTDESFVATFGVHNYKRWCDIVTNLTNEQTALVINQEYYGVPERDQARENQLTSYNVANAKGRRFAIEYTVTEGDNLKANLIIG |
| 4 | ABL4 | *A. bisporus* | MAMPRIFSPIINIFVFILTASYASAYLIPTPGVSAARPQLQQWSREQKYYQIHPMYDETKCLDVRQSGYKDGTPVDIFECNGTPAQNWSLQPGTTKVKVAGKNFCLDAGKAPTNGTKLKIWTCYPNLPAQTWYYTDDKRISLKDSGFCMDLTGGSNENYNPVQVWKCTNGNANQIWTV |
| 5 | ABL5 | *A. bisporus* | MYSLNGLVAATLLLSNGLGGLASPLVPRQKVGPVGTLHPISSSEKCIDVRGAAFENGTAVQIFDCNGSDAQNWMIQRGVAGHIEVEGTGFCLDAGSSPANGVGMKIWQCFDNLPAQQWTYNSDNQIKLADKDFCLDLTDGNSGNSNQLQIWECSTGNRNQVWLEGPPGNGESTPPPPPPPTSRALHSNVDGSKCMDVEGAVFANGTPVQIFDCNGSNAQQWTLTDGNTKVQVAGTNFCLDATSANPANGAGMKIWECFDGLAAQSWTYTDSHHLQLQSSAKCIDMTNGDTTNGNQLQVFDCFDGNNNQIWN |
| 6 | ABL6 | *A. bisporus* | MPPRLEQSSIMVQSLNGLGVAAILLFSSGFGALASPVAPRQIGAVGTLHPIASSGKCVDVRTSVFENGTVVQIFDCNDSDAQNWRIQRGVAGPIEVEGTGFCLDAGSSPALGVGMKIWQCIDNLPAQQWLYNNDNQIILANQDTNFCLDLTDGNLANTNQLQIWECATGNENQVWLEGPPGNDDPTPPPPTSRPLHPNANGDKCMDVEGSVFANGTPVQIFDCNGSSAQQWTLTDGNTKVQVAGMNFCLDATSANPANGVGMKIWECFDGLAAQSWTYTDSHHLQLQSSPKCVDLTNGDITNGNQLQVFDCFEGNDNQIWN |
| 7 | ABL7 | *A. bisporus* | MTYTISIRVYQTTPKGFFRPVERTNWKYANGGTWDEVRGEYVLTMGGSGTSGSLRFVSSDTDESFVATFGVHNYKRWCDIVTNLTNEQTALVINQEYYGVPIRDQARENQLTSYNVANAKGRRFAIEYTVTEGDNLKANLIIG |
| 8 | ABL8 | *A. bisporus* | TYTISIRVYQTTPKGFFRPVERTNWKYANGGTWDEVRGEYVLTMGGSGTSGSLRFVSSDTDESFVATFGVHNYKRWCDIVTNLTNEQTALVINQEYYGVPIRDQARENQLTSYNVANAKGRRFAIEYTVTEGDNLKANLIIG |
| 9 | ABL9 | *A. bisporus* | MTYTISIRVYQTTPKGFFRPVERTNWKYANGGTWDEVRGEYVLTMGGSGTSGSLRFVSSDTDEIFVATFGVHNYKRWCDIVTNLTNEQTALVINQEYYGVPIRDQARENQLTSYNVANAKGRRFAIEYTVTEGIISRPISSSDKCFIRLPSQKS |
| 10 | ABL10 | *A. bisporus* | MTYTISIRVYQTTPKGFFRPVERTNWKYANGGTWDEVRGEYVLTMGGSGTSGSLRFVSSDTDESFVATFGVHNYKRWCDIVTNLTNEQTALVINQEYYGVPIRDQARENQLTSYNVANAKGRRFAIEYTVTEGDNLKADLIIG |
| 11 | ABL11 | *A. bisporus* | MMQESFARKVVLAAMIGALGVLAQQPDVPTYTGELVIQNIFDNGKCMTAASNSDGAAVTLQGCTGAASQKWVFQDGSVKVHGNKCLDVSSGADVNGTKMQIFTCSSGNANQQWSYDVWSKRLSWKGHNKCLDLSAGSTADGNRLQIWDCVWNPNQFWNTGYLSNSLPQTSQAEQFGTNNCGTSSDQNSKCQTAWINSADDFCVWAPPALEAIGSSERYEVAWCTKSGRGTRVIPDGTLKGVHFVETPDYVQITGQGDFTKINIPAGDAGGELDNRGADGRGNPIGGLVYGNSFGQGQQFHEWTNFMSDHEFCFRACRGPKAMELCNHVFDEMGCYWNMPANYDPGFENCEGVDAEPMGVYTVDGQRSTYTQGQSPTPPPHPAPSSSNCQALPTVSSSPVQARRALDRKFVPKFPEHTPAPSYF |
| 12 | ABL12 | *A. bisporus* | MAVNEINSGNRYTLRNVANTDAVLDLSGEDNRSLIGYEPHGGDNQKWLLEEVIGGWLLQNVASGLYVSVDASPDQDGDYANDTNVIGAEEKFIWHIWQEEGVENAFRDRVSVPNVRKSLDLSNHGAGPDVKIWGKWEGKNQLWYFEEA |
| 13 | AAL | *Auriculariopsis ampla* | MYSIAILALLPAVLAKPLPRQTTSTAGAVHIHPSDNTNFCLGAAGTGNGALVDIYDCTTKTSSTPAWVLDNDKKRFQLSGTQMCLDAGNNPSDGTAMKIWQCYDNLTQQSWAYNNEHFSVGSGAECLDLQDGRLADGATTQTWACSASNANQLWTTTAATATTTPSTPSASGQALHPNGSSGKCLNVAGDVANGTPVQIDDCDDSSAQKFVLSRGTTAVKLAGTDFCLDAGEDVGNGASMKIWQCYEGLAQQTWYYTEDNRIAVQGAGQCLDVSRGDLTDGNTMQTWICTDGNTNQIWN |
| 14 | BEL1 | *Boletus edulis* | MGGSGTSGVLRFKSDKGELITVAVGVHNYKRWCDVVTGLKPEETALVINPQYYNNGPRAYTREKQLAEYSVTSLVGTRFEVKYTVAEGNNLQADIIIG |
| 15 | BEL2 | *B. edulis* | MGGSGTSGVLRFKSDKELITVAVGVHNYKRWCDVVTGLKPDETALVINPQYYNNGPRAYVREKQLAEYSVTSLVGTRFEVKYTVAEGNNLQADIIIG |
| 16 | BEL3 | *B. edulis* | MSYTITLRVFQRNPARGFFSIVEKVNWAYGGTWSEANGTQTLHMGGSGTSGVLRFKSDKELITVAVGVHNYKRWCDVVTGLKPEETALVINPQYYDNGPRAYVREKQLAEYNVTSLVGTRFEVKYTVAEGNNLQADIIIG |
| 17 | BEL4 | *B. edulis* | MLSATRCFAALYFAFSLSLLSTLPSVYAAVDTKVANRTIERTVQLRTHSIHPPYIDEDLQNRWWDFGADAYINTNKHIRLTKATPSQMGWLWSRLPLSSTNYVIEIEFKVSGASSHLYGDGMAIWLTTERTQPGPVFGNKDKFEGLGIFVDTYANARHTFGFPRIVAMLGDGQTPYDHEHDGDANSIGSCSANVRRTNVVTKLKMTYVKDAYLDVKVHYKAWDDWTDCFRIDGISLPLSPYLGVSALTGEVFDAHDVISITTHSAVLSSQDAPLNKLTSSGGLFGSRHTDGSARGSSWLGWIIKLLFLAGVCVGGAYGYKAYKSRGRHGVLSGLGMDDRRGYGSRRRF |
| 18 | BEL5 | *B. edulis* | MSSVQANNSYSLTNVKGGTCLDLSGGDNVSIIGYGYHQGPNQTWTFQDAGNQTYNIKSAGSGQYLSVAGEPSDGQRVVASGSPYAWRVEDQQGVDGGVRLLPANNTKFCVDLADHGSSTPGTSVQLWSRWEGQNQIWKVQKVQ |
| 19 | BEL6 | *B. edulis* | XTYSITLRVFQRNPGRGFFSIVEKTVFHYANGGTWSEAKGTHTLTMGGSGTSGVLRFMSDKGELITVAVGVHNYKRWCDVVTGLKPEETALVINPQYYNNGPRAYTREKQLAEYNVTSVVGTRFEVKYTVVEGNNLEANVIFS |
| 20 | BEL7 | *B. edulis* | MSYSITLRVYQRNPGRGFFSVVEKTVFHFANGGTWSEANGTQILTMGGSGTSGVLRFKSDKELITVAVGVHNYKRWCDVVTGLKPEETALVINPQYYNNGPRAYAREKQLAEYSVTSVAGTKVEVKYTVTEGNNLQADIIIG |
| 21 | BEL8 | *B. edulis* | MSYSITLRVFQGNPARGFFSIVEKTVFHFANGGTWSEANGTQILTMGGSGTSGVLRFKSDKGELFTVAVGVHNYKRWCDIVGGIKPEDTALVINPQYYNNGPRAYVRERQLAEYSGTSPAGTKVEVIYTVPDGNNLQANIIIG |
| 22 | BEL9 | *B. edulis* | MSYTITLRVFQGNPARGFFSIVEKTVFNFANGGTWSEVNGTQVLTMGGSGTSGVLRFKSDKGELFTIALGVHNYKRWCDVITGIKPEDTALLINPQYYNGGFRAYQREKQLAEYGGNGAAGTWVQVIYTVTEGNNLEANLIIK |
| 23 | BEL10 | *B. edulis* | MSYTITLRVFQRNPARGFFSIVEKVNFAYGGTWSKANGTQTLNMSGSGTSGVLRFKSDKGELFTVAVGVHNYKRWCDVVTGLKPEETGLVINPQYYNGPRDYMREKQLAEYNVTSLVGTRVEVKYTVADGNNLQADIIIG |
| 24 | BEL11 | *B. edulis* | MLSATRCFAALYFAFSLSLLSTLPSVYAAVDTKVANRTIERTVQLRTHSIHPPYIDEDLQNRWWDFGADAYINTNKHIRLTRAVPSQMGWLWSRLPLSSTNYVIEIEFKVSGASSHLYGDGMAIWLTTERTQPGPVFGNKGPSTRISARWPRAHVIPPFADKFEGLGIFVDTYANARHTFGFPRIVAMLGDGQTPYDHEHDGDANSIGSCSANVRRTNVVTKLKMTYVKDAYLDVKVHYKAWDDWTDCFRIDGISLPLSPYLGVSALTGEVFDAHDVISITTHSAVLSSQDAPLNKLTSSGGLFGSRHTDGSARGSSWLGWIIKLLFLAGVCVGGAYGYKAYKSRGRHGVLSGLGMDDRRGYGSRRRF |
| 25 | BEL12 | *B. edulis* | MSTDQANVILFRTQTTPAEALTIFSSTGLRVIFSVSSWTDTVPQPTLGGGRATLYLLERPQVYFPPLPSLAPFGVPTPDDWRALWAAWDLVTLGMIPSEMLHRKPIDLRHKCLFYIGHIPTFLDMLMSKALNEPNTEPKNFTLIFERGIDPHVDDPEHCHRHSEVPNKDEDWPTLGSVLAFRDRVRARLLRLYDDVAAGRRTLDRHIARMLAMTLEHEGWHVETLLYMLIQCAGTGTLPPPGFAPPPWSALAAAWDASVRAPGTPTVTLGPATVMLGHYDCEGDDLKPELTHRVGDHEFGWDNESPARAVAVGRVRMEWRPVTNEEYLVFWNAGGKSQVGFPPSWMSSENEIQVRTVYGPVPLSVARHWPVLTSYDGLSAYAKSKGGRLPTEPELRLFLDTYDVSYTSGANVGFRNWYPVPATAGLDEHDGKGTNGGVWEWTSTFLNDHEGLFPTTHFPGYSTDFFDGKHHIALGGSYATMPRLAGRRTLRNFYQHNYPYPWVGARVVYDA |
| 26 | BEL13 | *B. edulis* | MSSVQANNSYSLTNVKGGTCLDLSGGDNVSIIGYGYHQGPNQTWTFQDAGNQTYNIKSAGSGQCLSVAGEPSDGQRVVASGSPYAWRVEDQQGVDGGVRLLPANNTKFCVDLADHGSSTPGTSVQLWSRWEGQNQIWKVQKVQ |
| 27 | CAL | *Cantharellus anzutake* | MFRRLTALATVLAVCARRCLSEITLENVESVTESTLNLHTHSIFAPYVDQDLQNRWFSFGADAYINTNKHIRLTQDRPSQTGWLWSRLPITVPNFQIEVEFKITSSSGHLFGDGMAIWLTTRRAESGPVFGFEDKFEGLGIFIDTFANERHSYGFPRILGMIGDGNTHYDNGKDGHEQAAGACTAQVRQQDIATKLRITVLKNHFVNVEVHYRGWDEWTPCFTIEKPKLPPAPFLGVTALTGEVSEAHDVISITASGLIINADKLRGPETPIRQKHRSSSSLFGGFFRFLLKLVFLGIILAVAYVGYIAYVKKYGSGKVPWDAKRF |
| 28 | CNL | *Clitocybe nebularis* | MSITPGTYNITNVAYTNRLIDLTGSNPAENTLIIGHHLNKTPSGYGNQQWTLVQLPHTTIYTMQAVNPQSYVRVRDDNLVDGAALVGSQQPTPVSIESAGNSGQFRIKIPNLGLALTLPSDANSTPIVLGEVDETSTNQLWAFESVSAV |
| 29 | CMI1 | *Coprinellus micaceus* | MTSEIGVTQNLPVPVKRVGHSDIIGFGQDGVVIVRNTFRPKPRLVVRDFGYNAGGWRVERHVRLVGDTTGNGLGDIIGFGDLGVIVARNNGDNTFGPPGLALSDFGAVAGWTNTKHIRYVADLRGTGAVDIVGFGDEGVFVSLNNGNGNLAPAKLALKDFGYAAGGWRLDRHLRYLADTTGDGLPDIVGFGDSSVFVAVNKGDGTFHPVKPVLNDFTYESGGWRVENHPRTVADLTGDGRGDLVGFANDGVHVALNIGNGTFQPPRRVEENFGWEHAWRVQDHFRFIADTTGDGRGDIVGFGDAGVWVARNNGDGTFASRTLVLGSFGTQQRWLAGKHPRFFADLTGDGAADIVGFGEDAIWVSYNDGHGNFSPAEKLTDNLGFNNGEWGIDKAVRWVADLFH |
| 30 | CMI2 | *C. micaceus* | MTSEIGVTQNLPVPVKRVGHSDIIGFGQDGVVIVRNTFRPKPRLVVRDFGYNAGGWRVEKHVRLVGDTTGNGLGDIIGFGDLGVIVSRNNGDNTFGPPGLALSDFGAVAGWTNTKHIRYVADLRGTGAVDIVGFGDEGVFVSLNNGNGNFAPAKLALKDFGYAAGGWRLDRHLRYLADTTGDGLPDIVGFGDSSVCVAVNKGDGTFHPVKPVLNNFTYESGGWRVENHPRTVADLTGDGRGDLVGFANDGVHVALNIGNGTFQPPRRVEANFGWEHAWRVQDHFRFIADTTGDGRGDVVGFGDAGVWVARNNGDGTFASRTLVLGSFGTQQGWLAGKHPRCFADLTGDGAADIVGFGEDAIWVSYNDGHGNFSPAEKLTDNLGFNNGEWGIDKTIRWVADLFH |
| 31 | CMI3 | *C. micaceus* | MNTFVCDPGTPPSTEQSPLSTPTMKSEVGITQELPVPVKRVGAADIIGFGQDGVTILRNTFRPEPRLAIKNFGYNDGWHVEKHVRIVGDTTGNGLADIIGFGDAGVLVSRNNGNGTFGPPGLALRNFGADAGGWTNAKHVRYAADLRKKGFVDLIGFGDAGVFVALNNGNGNFAPAKLVLKDFGYVVGGWKVGRHLRFLADTTGDGLPDIVGFGESSVFVSFNNGDGTFQPVKEVINDFTHATGGWRMDKHIRTVADLSGDGRVDILGFGEDGVYVALNIGNGTFQPVKHAIQGFGYNNTWRVGIHPRFVADTTGDGLGDIVGFGSGGVYVARNNGDGTFATATIVLYEFGYDHGWRVDKHPRFLADLTGNGAVDIVGFGEDAVWVSYNDGKGNFPAAVKIATEFAFNDGQWAVDKTVRWVSNMF |
| 32 | CMI4 | *C. micaceus* | MKSEVGITQELPVPVKRVGAADIIGFGQDGVTILRNTFRPEPRLAIKDFGYNDGWRVEKHVRIVGDTTGNGLADIIGFGDAGVLVSRNNGNGTFGPPGLALRNFGADAGGWTNAKHVRYAADLRKKGFVDLIGFGDAGVFVALNNGNGNFAPAKLVLKDFGYVVGGWKVGRHLRFLADTTGDGLPDIVGFGESSVFVSFNNGDGTFQPVKEVINDFTHATGGWRMDKHIRTVADLSGDGRVDILGFGEDGVYVALNIGNGTFQPAKHVIHGFGYNNTWRVGMHPRFVADTTGDGLGDIVGFGSGGVYVARNNGDGTFATATIVLYEFGYDHGWRVDKHPRFLADLTGNGAVDIVGFGEDAVWVSYNDGKGNFPAAVKIATEFAFNDGQWAVDKTVRWVSNMF |
| 33 | CMI5 | *C. micaceus* | TFFGVVQATDDKTKFGNNTIERTVQLRTHSVYAHISTKIFRTGPLTDGTQNTNKHIRLTQNKPHQMGWLWSACIQVSGEPTHLYGDGMAMWLTTERTQPGPIFGNKDYFNGLGIMLDTYANERHTYGFPRVVGVLLDGKTQYDWGNDGDSQALGGCSVNFRRTNVATKLKLTYLKGSFLDVKVQYKAWDDWTDCFSVPNITLPSNPFVGFSALTGDVSDAHDIISVTSYSAILSPPEAPRNTIKKNKIWQRNPYPVDGSSQGTTWFALIFKLALFGGVCAGGWYGYKEYQRRQRYGGGGFGNLGGGYGLGGPGSAGFYANAKRF |
| 34 | CMI6 | *C. micaceus* | MGLPLPLLPLEVSLIAPPLNLTPLLLRWNLEADSELIAWQPTPPPPNNGWYPPPPKGQTVQLHPNYNWKKCLDVKDGNFYNGAPVDIYDCNGGAGQNWVINQGNTRVQVAGQNFCLDAGDWPNNGSKMKIWNCYDGLASQSWFYTDDKRIALKDKGFCLDLTNGDTCNGNVMQIWSCTNWNANQIWST |
| 35 | CMI7 | *C. micaceus* | MITTLKPLALLLALATPLLADSTVEKRQSGYATQFIIRNSCPAPVNLYIGSQFDSTLPVGGNTTKFSYIYTELFFTDANGGNPNGAGTTRAGFFDDGYYYIVKDEVGPLNTGLSIIPARTPSNGYCVSATCLQNNCTTAFNSIPARFPPVNEFSPPPTPPYYRCPELNTTYSITFCPDGGWPLKGSFRIKPGYGAVNKCLEVRGGVVANGTPVQIYDCNGTPAQEWYVGRGRTKVQLAGTNFCLDAGAVPANGVGLKIWECYDNLPAQQWYFTSDNRIALEGQGQCLDLPSGDLSNARQVQTWQCTDNDFNQVWSI |
| 36 | CMI8 | *C. micaceus* | MITTLKPLALLLALATPLLADSTVEKRQSGYATQFIIRNSCPAPVNLYIGSQFDSTLPVGGNTTKFSYIYTELFFTDANGGNPNGAGTTRAGFFDDGYYYIVKDEVGPLNTGLSIIPARTPSNGYCVSATCLQNNCTTAFNSIPARFPPVNEFSPPPTPPYYRCPELNTTYSITFCPDGGWPLKGSFRIKPGYGAVNKCLEVRGGVVANGTPVQIYDCNGTPAQEWYVGRGSTKVQLAGTNFCLDAGAVPANGVGLKIWECYDNLPAQQWYFTSDNRIALEGQGQCLDLPSGDLSNARQVQTWQCTDNDFNQVWSI |
| 37 | CMI9 | *C. micaceus* | MAEKIQTGRTFIIRNIQSGTVIDISGTDNRSIVGYPQTNGLNQRWTVNWTGGGWTLRSGSGNLYLALAAVPGDGVHLVASPSPFEWHIWHDEVFPGTYRIFAPRTVYNLDLWNYGDQKPGTAVTLWHKWNGAHQLWDFVQGE |
| 38 | CMI10 | *C. micaceus* | MAVVERGQRYKIVNAKSGTVLDLSSHPPPSSTYTALTTCLSLVNGWNFHGRDNQIWEASEEHGFWHFKNVASGKYLAPKSTQYKDGIKVIASDTRFNWHIWPDKKNPNTLRICIPDTVFNVDLSNHGDAKGGTPVELWGRWEGTNQTWKFE |
| 39 | CGL2 | *Coprinopsis cinerea* | MLYHLFVNNQVKLQNDFKPESVAAIRSSAFNSKGGTTVFNFLSAGENILLHISIRPGENVIVFNSRLKNGAWGPEERIPYAEKFRPPNPSITVIDHGDRFQIRFDYGTSIYYNKRIKENAAAIAYNAENSLFSSPVTVDVHGLLPPLPPA |
| 40 | CCL4 | *C. cinerea* | MIKSLLATFVSLAAFSTIVEAQLPEYTGELLLRPSINDNKCLTASALHDGAPVVISTCTGAASQKWVFTGNNGLVQTANNMCLDVTDGVNADGTKLQVWTCSPGNPNQNWWYDKWSNTLSWTDRWRCLDVTDGIQDDGNQVQVWGCWDKNPNQIWSTGYMPNALPYQSQNGQYGTNQCGGGNDQNSNCQTSWINSATDFCLWGPPWHGPVGNTERVAVAYCTKNGRGTRSIPDGTLTGVHFVRTPEYVQITGTGDFTNLNIPAGDDGGELDNKGADGKGNPIGGLLFGNTFVNGLQYNEWTQFISDREFCIRACIGPRAKQLCNHIYDVMGCWWNMPANYNSGVFEECEGDPATPMGVYDGTSTWYQGVSPTPPPHPAPASSNCVTLPTVGVSDMLRRRGVDSVGGFETRKVPEFPGATPAPVAR |
| 41 | CCL5 | *C. cinerea* | MRLSALYLALHSSALTLAASLLETRQTTPLTQYIVENQCPSAINLFIAGQLDSSIPSQGNTTKFLPANAGFFYTDANGGNSNGLRTLRAGFYGEQFYYYMVKDPDYVNVGMNVAPQGVPSRQGYCVSVGCSDVNCPTAFSSPPTRFPPSGSTAPNPPFYQCPIANTTYRITFCPGGQWPGPETWRIHPGFNTAKCLDVRGAVFSNGTPVQIYDCNGTAAQNWILNRGATSIRLNGTNFCLDAGSNPANGVGMKIWQCYDNLPAQQWFYRDDNRITLQNQGRSSIILICGVAP |
| 42 | CCL6 | *C. cinerea* | MRFLLKCLPTFALLGGALTQNTFSARIRNNCGNTMRLFIDGVDTGPLLPGGDHFANLGLDTVIYNTLNGGNPDGSATTRVFFWGERFYYYIVKDAGVSNVGVSISVPEHPKSVDGLCTTITCEPATCSSSEAYSEKQAFTENAIPVSSSPALPLRSCPFGGARYVVEWCPNGTMPPGWEQNGQEIQLVVPGDRIWCVDVRAAAFTPGTPVQLYRCNGTPAQKWLVPSPATTPGEVRLFGTDLCLDAGSNPHNGGLLTIQQCDGRPSQVWVYDDTNGRYTFSTIGGQCMDDTNGLKEDWVQLQTWGCGPPENPNQKWLASVAIAP |
| 43 | CCL3 | *C. cinerea* | MNLLAFLSTSLLLLPLHTLASQSAQVPIPGATQAENTYVSRIHPSAAVWKKCIDVRGGELELRTPIQVYDCNGTPAQAWVYDGRTLHGTLLRVSGTPYCLDAGDPPFTNGKKVHLWECIHGIPAQIWRYDYTGRWYLGDSGYCLDLTDGKLDNGNQLQIWECNDGPNQKWIIDL |
| 44 | CCL1 | *C. cinerea* | MDTQAKPPAGRYVIYNRVLSPNGEKLALTYPGYQREALTLTPLHHSANQIWILNNWDATTSSLTPETARNTQVGWGSGNVPIVLPPGNYVWTLASSAGGYIIQDGGKTVYWNAGEAVANTKVSIGGDNGLQARWIFERV |
| 45 | CCL2 | *C. cinerea* | MDSPAVTLSAGNYIIYNRVLSPRGEKLALTYPGRQRTPVTVSPLDGSSEQAWILRSYDSNSNTWTISPVGSPNSQIGWGAGNVPVVLPPNNYVWTLTLTSGGYNIQDGKRTVSWSLNNATAGEEVSIGADATFSGRWVIEKV |
| 46 | CML1 | *Cubamyces menziesii* | MTFSKDGIYHIEHAHVAVRIALAEGSNIDETPVIAWSIYDDYLDHMWLIQSVSGEADTYTIRNTVAGTYMDLTKGLTENGTAIIGFQKVKPGDNQKWIIKKESSATGYWKIQNKATFVDLLNGGSSNGTKIVGWEGSWDDSTSLGHQHWTFSPQSLPGHEIHTILKANTYLRQDFKSYETDGMYLILSRARLQEIWNKSGLSSRKWREEIFDCDDFSFVYKSEVAKWGDNHFKADGFAIICGIMFGTNSNAAHAYNWMIDPADHSDIIFFEPQRNVFKEDPGYKAYFGVF |
| 47 | CML2 | *C. menziesii* | MTFNKDGIYRIEHAHVAVRIALAAHSSEDGTPVIAWNVNDDYLDHMWLIKSVPDEPDTYTIRNTVAGSYMDLSASSAEDGTPIIGFHKTDPGENQKWIIKKETSGTGYWKIQNKASKTFVDLLDGGSSDGTKIVGWQGSWEDGPPPGHQHWTFSPQSLLGHEVHTILKANPYLRQDFKSYLADGMYLILSRERLQDIWRNSELPSRHWREEIFDCDDFAFVYKSAVAKWGDEQFKADGFAIVCGVMFGTRVHEGEVDGHAYNWVVNPEDHSAIVFFEPQNNTFMDDPGYKAYFGVF |
| 48 | CML3 | *C. menziesii* | MTFSGDGIYRIEHGRVPVRIALARHSSEDGAAIVVWNLKDEYLDHLWLIKSVPGQADTYTIRNTVGGTYMDLKKSSRDNGTPIIGWHWTGNDNQKWIIKKETSGTGCWKIQNKAAESFIDLYDGESADGSKIVGWQSSWNDGPPGKSLGHQLWTFSPQSLFGHEIHTILKTNPYLRQDFKSYISDGMYLILSRTQLQEIWSKMGLTSKDWRSEIFDCDDFAFTFKTEVAKWGKNEFNANGFAILCGVMFGTRGSSGHAYNWMIDPSDHASIIFFEPQNGSFMYNPGYNAYFGVF |
| 49 | CSL1 | *Cyathus striatus* | MSYKITVRVYRTNPNAFFRVVEKTVWKYANGGTWDEANGQHILTMGGSGTSGTLRFVADNGENFIVALGVHNYKRWGDIVTNLSNEQTGVVINPEYYSVKEREQARERQLASYNVNNAKGRNFSINYTVADGNDLRCNVIIG |
| 50 | CSL2 | *C. striatus* | MSYKISVRIFQTNPNAYFRVVEKTVWNYANGGAWDTVNDEYVLTMGGSGTSGTLRFLADSGENFIVAVGVHNYKRWGDIVTNLSNDQTGIIINGEYYNVKEREQAREKQLSNYNVKNAKGRNFAINYTVADGNDLRCNVIIG |
| 51 | CSL3 | *C. striatus* | MSYKITVRVFQTNPNAFFRVVEKTVWKYANGGTWDEVNGEHVLNMGGSGTSGTLRFVADNGENFIVAFGVHNYKRWGDIVTNLSNEQTGVVINPEYYSVREREQARERQLASYNVKNAKGRNFAINYTVADGNDLRCNVIIG |
| 52 | CSL4 | *C. striatus* | MSYKITVRVFQTNPNTFFRVVEKTVWKYANGGTWDEVNGQHVLNMGGSGTSGTLRFVADNGENFIVAVGVHNYKRWGDIVTNLSNEQTGVVINPEYYSVKEREQARERQLVSYNVKNAKGRNFAVTYTVAEGNDLQCNVIIG |
| 53 | FAL1 | *Flammula alnicola* | MSEILYIPPTGISFRLINYNSGYVIVSRNSDPQVLHYDPAKGTHDEQFFQLLHGTGGHSGEVAIRGNVTGKFLYVPADKDHSVYHTSGDGSAEYRFTLEPGSGKLANYFRLRDIATNQVIYSRRSPDPTFHNYNANKTRYDDQYWTYLFEDVEVVDVHYNLDAGKILSTTPIVVGNQTLKNETDEEQTMSLAINDTESQTSTFQYRVGFTVSVGMKFKAGIPLVADAGFSINTSLTNEWTMGESTTYTKSYIVTFPVKAGPHQTIKAVSTVSRGVLEVPFTMILKSKATGFQVTMDGIWSGISTWDLRHAISKVVEGDYY |
| 54 | FAL2 | *F. alnicola* | MSYKITLRIYQTNPNAYFRVVEQTVYKGTWTEADGERKLSLNNSGVCGTIRLVADNGENFLVAIGIHDFQRWCDIVTNLGEDDTGLNINPQYCGARSGIREKKLSSYSISNVKGRKFSINFTVAEGNDLKANVIIG |
| 55 | FAL3 | *F. alnicola* | MTSNVISQNLPVPTARVGTADIVGFGTSGVIILRNSVNIQTFKVIANFGYDAGGWRVDKHVRLVADTTGNREADIVGFGENGVWISFNNGNNTFQEPKMVMAGFAYAADLRKTGRADIVGFAHDGVLVSLNNGDGSFAPAKLAVADFGYVAGGWRIEKHLRFLGDVYGTGMLDIIGFGDNQVVISKNNGNGTFHPSHGVVNGMCYSAGGWLIEKHPRFVADLTGNGMVDLIGFGDAGVSVALNNGNGTFQASKLVLSEFGYDGGWRVEKNPRFIADLTGNKAGDIVGFGNEGVYVALNNGDGTFQPAKKVINSFAYDVGGWRVEKHPRFPVDLTGDGCADIIGFGDNSVFACYNDGKGNFGPMQKLVDQFAYNGGEWSLEKTGSRIWILWVNHPAYPA |
| 56 | FAL4 | *F. alnicola* | MNVASVAFLLSFTSLFVGVAQAAADDKTRLGNNTIDRTLQLRTHSLYAPYIDQDLQNRWWDFGADAYVNTNKYIRLTRNRPSQMGWLWSRLALTPSNFVVEVEFKVTGDSSHLYGDGLAVWFTKDRAVEGPVFGSKDEFTGLGIFLDTYANEKHSYSFPRIVGILGDGKTKYDFANDGDGQDVGSCSANFRRTNVATKLKITYVKDAFLDVKIQYKAWDDWTDCFYVEKLALPTNPFLGFSAMTGDVSDAHDIISVTSYSAIVSQPDAAPNKHRKTSIFGSAHPDGSSGTWLGFFFKLFLLAGVVAGGYYGWQEYQRRQRYGGFGGGGGMGGYGMRSAGVGGGFGDYGGKRF |
| 57 | FAL5 | *F. alnicola* | MRYLTSFLSAATAAAAVRAALPPANEFVQLQPITRGSFPRTPCLTVFSAANGSALVINDCSAATQQRGFQVVEGGATTGEGTPGAIKIFNSFCLEVTGGADLDGTKVEINSCVAGDANQLWEWNADGTVVWSGTNKCLDLTNGDLENGNQIQIWTCFAGNTNQQWTSNTLANPISEVFIASQPQFCMAADSSENGSPVFITSCSDTTALKVWTVPQIGHGNSGSYKLAFGPGGSPPLKCLDLTNGNTTPGTKLQLWDCDPANINQDWIPTGTIRWEGTGLAIPMCVDLTDGITTRGNPLQIWNCTGNSNQIWDDVAPPQTAV |
| 58 | FAL6 | *F. alnicola* | MRYLTSFLSAATAAAAVRAALPPANQIVQIQPITRGSFPRTPCLTVFPAANGSAVLINDCSTSTEQRGFQVVEGGATSGEGTPGPIEVFNTFCLEVTGGANVSGTKIEINSCVAGDPNQLWEWNSDGTVVWSGTNKCLDLTNGDLDNGNQIQIWTCTAGNINQQWTANPLVNPISEVVNAAQPELCMAADSSENGAPVFITSCSDTTALKVWIVPQIGHGNSGSYKLAFGPDGSAPIKCLDLTDGNTTPGTKLQLWDCVGNENQSWLPTGIIRWQGTGLLNPMCVDLTDGITTRGNPLQIWNCTAGNSNQFWNDVAPPQTAV |
| 59 | FAL7 | *F. alnicola* | MRYLTSFLSAATAAAAVRAALPSANQVVQIQPITRGPFPRTPCLTVLFPAANGSALIINDCTTSSQERGFQVVEGGATTVGKGTPGPIEIFNTFCLEVTGGADVSGTKVEINRCVTGDPNQLWEWNSDATVVWSGTDKCLDLTNGDLDNGNQIQIWTCTAGNPNQQWTANTLANCIALYPGLSMPDNVIIFSYLIQVKPQFCMAADSSENGAPVFITSCDDTTALKIWSVPQVGHGNSGSYKLAFGPDGSAPIKCLDLTDGNTTPGTKLQLWDCVGNLNQDWTPGPTDTIRWEGSGVNDSLCVDLTDGITTRGNQLQVWTCTAENTNQLWNDTAPPQNVV |
| 60 | FAL8 | *F. alnicola* | MHYLTYFLSAVTAAAAVRAALPPANQVVQIQPITRGSFPRTPCLTVLYPAANGSALIINDCSTLTQERGFQVVEGGATSGEGTPGPIEIFNTFCLEVTGGADVSGTKVEINRCVTGDPNQLWEWNSDATVVWSGTDKCLDLTNGNLNNGNQIQIWTCTAGNINQQWTANTLANPISEVVIAAQPDFCMAADSSENGAPVFITSCNDTTALKVWSVPQVGHGNSGSYKLAFGPDGSAPIKCLDLTDGNTNPGTKLQLWDCVENLNQYWTPGPTDTILWEGSGEGDLLCVDLTDGITTRGNPLQVWTCTAGNTNQLWNDTAPPQTAV |
| 61 | FAL9 | *F. alnicola* | MTFINVLAFPLLFFLPLVLSSPLSPRAPWIPVYLACPSELTPSGGGSYRLVEASTFSQYYQCWYWDNDPTKGDDQFCWYDRKKLRRSTRHYRRTFFLSPDIPLPPNPQLIICFQECNFSSGLNRLQTWFFDGSTLKLVGTNKCLDVTDGNTSNGAKLQVWTCVDGARNQQFYHWDHVQLIVPEDHISWMHLPGKCMDLTDGVVQDDTRIQMWDCSYQNPNQKWSVLPALDE |
| 62 | FAL10 | *F. alnicola* | MRSSVFQALTAFAFLAVSVNAQGTPAFIGQLLLEPGINSAKCLNAASNTDGAHVTIQPCNGSTAQKWTFSGGNVKIFGNKCLDVTDGKNADGTKMQIWTCTTNNANQKWDYNRWTNRLTWLNKNKCLDLTDGRQTDGNQIQVWTCAGGNINQVWNAGYSISNLPQTSQADQYGTNNCGTGSSSSSNCQTGWINSASDFCIWGPPYHATIGDIEAEAVAYCTKSGRGARTIPNGTLKGVHFVKTPEYVQVTGVGDFTKINVPKGDEGGELDNRGADGRGNPIGGLLYGNSFGSGLQYHEWTSFLSDNEFCIRACVGARATSLCNHIYDIMGCYWNIPASYSSGVYEDCSGNAALPMGVYGTSTWYQGVNPTPAPHPAPASSNCNALPTVSVSPALARRDNLVKRYVSPAFPGATPAPLA |
| 63 | FAL11 | *F. alnicola* | MLSILCTLLFTLLTIGHPLSPRQVDERTYVVHNNCPGQINLFVGGDLDAVLPAGGKFSEVASTFAGFWYTDANGGRYTGVGTTRAAFWGDSYGIIKDPGGINTGMTVVPRHAPTTEGFCTSIACEDSQCRQGFTGIPGFPNDTSPLPYHHCPFNNTTFDITFCPNGIFPANAGAEIHPNSDSAKCLDVRGANFANGTPVQIYDCNGTGAQKWFYVRGSTKVQVAGTNFCLDAGSSPASGVGLKIWQCYDGLAAQQWYYTNDNRIALEGKGQCMDLTNGVLANGNQVQTWQCTDFNANQIWNPF |
| 64 | FAL12 | *F. alnicola* | MLSSLLYSLLLLATPLVASPLAPRQTTRQYTIINKCPTAVNLFIAGALDSSMPVGGSVTRTLPVDAGMFYTDANGGDGDSQGTTRAGFFENFYYIVKDPSYLNIGMTVAPHGSTSSSGFCAVAECESVDCTAFPQPPTGFMVLPYRRVPTAGATIHPNFNTAKCMDVRGANFANGTPVQIYDCNGTPAQSWTIRRGSTKVQVTGTNFCLDAGTNPANGIGLKIWQCYDNLPAQQWYYTEDNRIALEGQGFCTDLTNGIQTNSNQLQTWQCTDNNVNQIWTI |
| 65 | FAL13 | *F. alnicola* | MLALASAILAVALAARSVNSERLFTIVNRCPDAVIPYINGQAQDVLATNSVTNQTFQDDFTGLIYTNANGGAANGAGSTRAGFFGPTNYYYIVGDAAHLNTGVGVVPKVIAANALESKFCAADTCDSTNCPGVYSQPPTSFPQPNVTAPPSPLFECPGASVGYTVTFCPEKTFPPPPGTVSIHPNGTTSQCLDVQGAQFANGTPVQIYDCNGTAAQRWVVTKGGNSTRVALAGTNFCLDAGTNPANGTGMKIWTCFNNLAAQTWMYTDANEIKLASDDLCLDLPNGSLTNGNRIQTASCDIAHLDQVWVSA |
| 66 | FAL14 | *F. alnicola* | MSTDPVQASHHHLFSLSAVRSIISTPITEQCTLHILERPSFMYKPLSYTIAHSLAPSVDGSAGNIPTLEEWKTLWANWDLITLQMIPSEMLHQKPIDLRHKCLFYIGHIPTFLDMLISKSIGGEATEPKYFWNIFERGIDPHVDDPDHCHNHSEVPEKDEDWPTIESIMGFRDGVRARLAQLYDDLATGKLALTRNIARMLVMTHEHEAFHVETLLYMLIQKAGCGGTLPPPGFIVPPWEDLARQWAAIPPPSTPTVLVGPATLAMGHDDSEGDDLTAASEPVLDVVGRTFGWDNESPARTIKVGAFKAEWRPVTNGEFERFCRGAGKDLVQMPKSWVQEAGGDVKVRTMYGFVPMHIAEHWPVLTSYDDLALYAKFKGGRLPTEPELRLFLDLYDVGHEGGANVGFRNWHPVPATMGLEAYGGKGSNGGVWEWTSTVFDTHDGLVPTKLFTGYSADFFDTKHQVSLGASYATIPRLAGRRTVRNFYQHNYPYPWIGARVVYDV |
| 67 | FAL15 | *F. alnicola* | MTAQVNSGSTYVITNVKAGTAMDLSAGDNTSVIGWPVHGAANQQWTMNWTGKAWTFKSGSTGQYLSIVGSPADGTRVVAAAESFEWHIWRADSDTNTFRIFVPFTHQNLDLYAGGNTTPGTPITLWYTWNGLHQTWKFTHGMNLCNSSLLVS |
| 68 | GLL1 | *Ganoderma leucocontextum* | MFFKGLLYCITLLPLGILAAIDDKGKLANRTIDRTVSLRTHSIHPPYIDQDLQNRWWDFGADAYVNTNKHIRLTRAKPSQMGWLWSRVPLTASNFVLELEFKISGDNNHLFGDGMAIWITKDRAQPGPIFGSKDNFDGLGLFLDTYANARHPYSFPRITAMLGDGKTPYDLDNDGEKNNLGACSANFRRTNVATKLKITYLREQYLNVKIQYKAWDDWTDCFTSYGTVLPLAPYVGFSALTGDVSDSHDVIGVTTYSAILSSPDAQRDKLTGSKKKLGLHTLTESSWSWTLIKLLAVAGILVGAFYGYRTYALRQGGGAGFGAVGRGMQAFGYDSKRF |
| 69 | GLL2 | *G. leucocontextum* | MDTVTPFRNIWTPGTYVLINARNKSVADLSAQDHDTVIGYPLHGGPNQQWEFIPSGNGYAIRSMRHPGVDLYMSVEGEARVKAPVVATGHHTVWTVEQTGDGLRISWPNSSLVFELADTVSGLRLMLKTLVPGELQQLWRFIRCSQPQPQPAEAIESAGGVEVVVESARAASDPATTETVTASESDDFITTTRTTTTTISTTVTEVIRTPKPRLQR |
| 70 | GLL3 | *G. leucocontextum* | MWTPGTYVLLNARGGTAMDLHGGDNTNVIGYPMHGEQNQQWEFIPSGHGYVIRCVRSSKAGHALYLTVEGGVRNNAPVVASAYPVAWSVEQTEEGIRISWPNSNFVFDLADWGDSTAGTKIQLMPLIPGELCQLWHYTRCAPADRDEKGMEIEVQSARAVSPPATTDTVFVSETHDFVTTTRTTMTSVITTVTEVTSTPKALLRQPAQQQPQPQRRSISYM |
| 71 | GLL4 | *G. leucocontextum* | MWTPGTYVLLNARGGTAMDLHGGDNTNVIGYPMHGEQNQQWEFIPSGRGYVIRCVRSSKAGHALYLTVEGGVRNNAPVVASAYPVAWSVEQTEEGIRISWPNSNFVFDLADWGDSTAGTKIQLMPLIPGELCQLWHYTRCAPADRDEKGVEIEVQSARAVSPPATTDTVLVSETHDFVTTTRTTMTSVITTVTEVTSTPKALLRQPAQQQPQPQRRSISYM |
| 72 | GLL5 | *G. leucocontextum* | MVESGRTYKLVNAKAGTVLDLSGADNRQTIGYGYHGGDNQKWALEQEDNQWLLRNVASGLYLSVEGLVEDGTSVLATGEHFKWDIWPDEEDSSTFRLFVPNTQFNMDLSDHGSAVPGTHVVIWTKWHPGKNQTWRFEEVPGIASDYLCAWASSGVAACVGAYHYWEFRLRAGATSAHYFYVFLLLATSRLFRGSLE |
| 73 | GLL6 | *G. leucocontextum* | MQLENGKTYKVTNAKGGTVLDLSGGEDQSPITGYNFSGNDNQKWVAEQQDQNWRFKNPATGLFIGYSGDPNNDTPIKAMRNPTLWDVRPDQENPDAFRAFVAGTEYCLDLSDHGNSTPGTPVTLWKKWGGANQTWRFEEV |
| 74 | LAL1 | *Lactarius akahatsu* | MSQGQGYSIKVLIFQTNPHQNEFFRVVEKTVWNYANGGTWSETDGCDILRMGGSGTSGALRLLSNKGEGCIITLGVHNYKRWGDIITNLKNDQTACVINPQYYSKDFPDREKQRERQLTSYEVADLQGRKFSFEYVVTDGNELTVRVVIA |
| 75 | LAL4 | *L. akahatsu* | LQSHKRWGDIVTNLKNDQTACTINPEYYSNEHANRQRQREKQLTAYEVALVADLQGRRYSLEYYVTEGNVLEVRIVIF |
| 76 | LAL2 | *L. akahatsu* | MGRSEELIPSQDKMAIDQDCVDLGVLCVSVCDALKRATDGKKLEDLNGFVLPALRAGLADIVDFGQDGVVILRNGFNTTGYNAGGSRIEEHVRLVGDVTGDGADDLVEFGEAGVLVSTNNGDNTFTSPVKLVLKDFGYDAGGWRVEKHIRYLADIRGVGRSDIVGFGHGGVIVSKNDGNAKFNPVYLALGDFGLPHRVARRNNGDGTFAPAQAVISGTFCYDGGGWRIEQHPRFIADLTGDGKVDIIGCGDAGVYVSLNKGDGTFGPINLVANIFGTVQGWKVDKHPRFIADLTGDKRGDIIGFGEAGVYVAYNNGNGTFQPGKLVLSDFGVQQGWQVNKHPRFVVDLTGDGRADILGFRENSVFVAYNDGKGGFPSVKTSVDKTVRWLANLDQASCKIT |
| 77 | LAL3 | *L. akahatsu* | MILAKLLLLVSSLCSASVLASDYDGTRIANRTIDRTVQLLTHSLFAPYIDQDLQNRWWDFGADAYVNTNKHIRLTRNKSSLMGWLWSRLPLTSHNWIIEVEFKISGGTSHLYGDGLALWITTERAQPGPVFGSKDHFTGIGIFLDTYKNDLHSEYPFPRIIAMNGDGKTSYDLAKDGVPNMIGECAADYRRSSVATKLKVIYVKDTVLDVKIQWKGWEEWSNCFTLNDISLPQSPYIGLTAMTGDVSDAHDIISVSTSSAILSSAETPRDKLQSTESEAGWFSFSFVRFLLFGGAVAGIWFGWRTYGQSGSKRDQAFWCALGTSTMDTTSVHAFSVTFL |
| 78 | LDL1 | *L. deliciosus* | MSIKSGQRYKITNQLTELAVDLNCADNKSIIGYNFHGGENQQWVLDVQVNGQWIIRSVNLQNLKYLGVENAPNNGTHLVGLDHPQFWDIEILPGSEDPTKLSVKICQTVHGTRFAADYPQEKPPVGADLQLWTAWGGKNQIWVLEECESRFDEVW |
| 79 | LDL2 | *L. deliciosus* | SIKSGQRYKITNEQTKLVVDLSGVNNKSVLGYNFHGGANQQWIIEQQVNDQYTIQSVAHHKYLGVEKTPGNGTHLVGLDKPQFWDIEIQPDSKDATKLSAKFCQWARGTCFVADYPVEKGAATDLQLSSALEGKNQVWVLEEFLKC |
| 80 | LHA1 | *L. hatsudake* | MSQTNQGYSIKVLVFQTNPHQNEFFRVVEKTVWNFANGGTWSEVDGCDILRMNGSGTSGALRLLSNKGQGCIITLGVHNYKRWGDIITNLKDDQTACVINPQYYSKDHPNMEKQRERQLSSYETTDLQGRKFSFEYVVSEGHDLTVRVIIA |
| 81 | LHA5 | *L. hatsudake* | MSTPLRRYSIKVLIFQTNPHQNEFFRVVEKTVWNYANGGTWSEVDGCDILRMGGSGTSGALRLLSNNGEGCIITLGVHNYKRWGDIITNLKNDQTACVINPQYYSKDFPDREKQRERQLTTYEVADLQGRKFSFEYVVSEGHDLTVRVIIA |
| 82 | LHA2 | *L. hatsudake* | MSREEGLQSLDPSAPERERSSIEFPTLRADLADIVDFGQDGVVILRNGFNVQSFLAIKDYGYNAGGWPIENHVRLVGDVTGDGADDLVEFGEAGVLVSINNGDNTFTSPVKLVLRDFGYHAGGWRVEKHIRYLADIRGVGRSDIVGFGDGGVIVSKNDGNAKFNPSYLALGDFGYHAGWRVERHLRFIGVATESGRPDIIGFGDKYVFIGRNNGDGTFAPAQAVINDFCYIAGGWRIEQHPRFIADLTGDGKVDIIGCGIAGVSASLNTGDGTFGPVNLVVNNFGTGQGWEVDKHPRFIADLTGDKRGDFIGFGEAGVYVAYNNGNGTFQPAKFVLSDFGVQQGWQVNKHPRFVVDLTGDGRSDILGFRENSVFVAYNDGKGGFPSVRTLTSELSFSGGK |
| 83 | LHA3 | *L. hatsudake* | MILGNLLLLVSSLCSASVLASDDDGTRIANRTIDRTVQLLTHSLFAPYIDQDLQNRWWDFGADAYVNTNKHIRLTRNKPSLMGWLWSRLPLTSHNWIIEVEFKISGGTSHLYGDGLALWITTERAQPGPVFGSKDHFTGIGIFLDTYKNDLHSEYPFPRIIAMNGDGKTSYDLAKDGVPNMIGECAADFRRSSVATKLKVIYVKDTVLDVRVLISSGEEWSNCFTLNDISLPQNPYIGLTAMTGDVSDAHDIISVSTSSAILSSAQTPRDKLQSTGSEAGWFSFSFVRFLLFGGAVAGIWFGWRTYGQRLLSSRAGNFGGGGGGLMWSDSKRF |
| 84 | LHA4 | *L. hatsudake* | MSESVKSGQRYKITNEENGLVLDISGANHRNILGWDFHGGDNQQWIAEKQDDGQWTIRSVWLQKYLGFEKTPEDRTPLVGLDKPQLWDIEVLSDSEDHDNPRVKLWLRGTLLVVEFPIEKADPGPTESDRQLQLLAARDGRNQVWVLEECAGRWSSGHDPPRH |
| 85 | LHE2_1 | *L. hengduanensis* | MALDVPTLRAGLADIVDFGQDGVVILRNGFNAQSFLAIKDYGYNAGGSPIEEHVRLVGDVTGDGADDLVEFGEAGVLVSINNGDNTFTSPVKLVLRNFGYGAGGWRVEKHIRFLADIRGVGRSDIVGFGDGGVIVSKNDGNAKFNPVYVALKNFGYHTGWRVERHLRFIGVTTGSGLPDIIGFGDNDVLIGRNNGDGTFAPPQAVINNFCYDVGGWRIEQHPRFIADLTGDGKVDVIGCGDAGVYASLNKGDGTFGPINLVVDNFGTGQGWWVDKHPRFIADLTGDKRGDVIGFGEAGVYVAYNNGNGTFQPGKFVLSDFGVQQGWEVNKHPRFVVDLTGDGRADILGFRENSVFVAYNDGKGGFPSVKTVTSELSFSGGK |
| 86 | LHE2_2 | *L. hengduanensis* | MYGTLTRRASLAEDGVVILRNGFNVQSFLAIKDYGYNAGGSPIEEHVRLVGDVTGDGADDLVEFGEAGVLVSTNNGDNTFTSPIKLALKDFGYDAGGWRVEKHIRYLADIRGVGRSDIVGFGHGGVIVSKNDGNAKFNPAYLALGDFGYHTGWRVERHLRFIGVATGSGRPDIIGFGDKSVFIGRNNGDGTFAPAQAVIRSFCYDMGGWRIEYHPRFIADLTGDGKVDIIGCGFPGVYVSLNKGDGTFGPVNLVVYNFGTLQGWEVDKHPRFIADLTGDKRGDIIGFAEAGVYVAYNNGNGTFQPGKFVLSDFGVQQGWKEVNKHPRFVVDLTGDGRADIVGFRENSVFVAYNDGKGGFPSVKTVNSELSFSGGK |
| 87 | LHE1 | *L. hengduanensis* | MSESIKSGQRYKFTNEENGLVLDISGANRRNILGWDVHGNDNQQWITERQDDGQWTIRSVWPQKYLGFENTPEDGTPLVGLDEPQLWDIEVLSDSEDHDNPRVKLWVRGTLLVVEFPIGKSDPRPIESDRQLQLLAARDGKNQVWVLEELY |
| 88 | LHE2_3 | *L. hengduanensis* | MSIKSGQRYKITNEQHKLVIDLSAADHKSVLGHTFHGGENQLWITEKHVNGQWTIQSVATGGYQKYLCVERTPDNGTHLVGLDQPQFWDIEILPVSTKRSVKLCQWVRGTYFVADYPQETGAGTNLQLWTACGGRNQVWVLEECESHLGEA |
| 89 | LPL1 | *L. pseudohatsudake* | MATLHSVGESNESRQSSFSIKVLIFQTNPGQNAFFHVVEKTVWNSADGGTWSEVDGCDTLRMGRSGTSGALRLLSNKGDGCIITLGVHEGKRWGDIITNLKNDQTACVINPQYYSKDHPNMEKQRERQLTSYDTSDLQGRKFSFEYTVSEGHDLTVRVIIA |
| 90 | LPL2 | *L. pseudohatsudake* | VPTLRAGLADIVDFGQDGVVILRNGFNAQSFLAIKDHGYNAGGLPIEEHVRLVGDVTGDGADDLVEFGEAGVLVSINNGDNTFTSPVKLVLKNFGYGAGDWRVEKHIRFLADIRGVGRSDIVGFGDGGVIVSKNDGNAKFNPVYVALKNFGYHTGWRVERHLRFIGVTTGSGLPDIIGFGDNDVLIGRNNGDGTFAPPQAVINNFCYDVGGWRIEQHPRFIADLTGDGKVDVIGCGDAGVYASLNKGDGTFGPINLVVDNFGTGQGWWLDKHPRFIADLTGDKRGDVIGFGEAGVYVAYNNGNGTFQPGKFVLSDFGVQQGWQHPRFVVDLTGDGRADILGFRENSKGGPDRGSVTRGGGGVSPKALKKFTFLATGGPVR |
| 91 | LPL3 | *L. pseudohatsudake* | MSESIKSGQRYKFTNEENGLILDISGANHRNILGWDVHGNDNQQWITERQDDGQWTIRSVWPQKYLGFENTPEDGTQLVGLDEPQLWDIEVLSDSEDHDNPRVKLWVRGTLLVVEFPIGKSDPRPIESDRQLQLLAARDGKNQVWVLEECESRLVKSNDRRG |
| 92 | LPL4 | *L. pseudohatsudake* | MSESIKSGQRYKFTNEENGLILDISGANHRNILGWDVHGNDNQQWITERQDDGQWTIRSVWPQKYLGFETTPEDGTPLVGLDEPQLWDIEVLSDSEDHDNPRVNRLWVRGTLLVVEFPIGKSDPRPIESDRQLQLLAARDGKNQVWVLEEYL |
| 93 | LPL5 | *L. pseudohatsudake* | MSIKSGQRYKITNEQTKLALDLHGINHKTIMGYHLHGGENQQWVIDEQVNGQWTIRSVDQQKYLGVEKAPDNGTHLVGLDQPQFWDIEILPGCEDATKPSVKYVTVRGTCFVVDHPLEKPPVGADLQLWTAWGGKNQIWVLEECESRFGEVW |
| 94 | LPL6 | *L. pseudohatsudake* | MSIKSGQRYKITNGQHKLVMDLFAADHKSVLGHTFHGGENQLWITEKQVNGQWTIQSVEHQKYLGVETTPNNGTHLVGLDQPQFWDIEILPDSKNPTKPSVKYWVRGTCFVADYPKNARTGANLQLWTTWGGKNQVWVLKERKCVLVKSGNSPIGS |
| 95 | MOA1 | *Macrolepiota procera* | MSTQVSSGQTYKITNVKAGTVIDLSGEDNKSIIGYPYHSGKNQQWTFNWTGKAWTLRSASSGSYLGIEGTPAGGTRLVAVNDPFEWHIWRDEANENAFRIFVPFTNYNLDLSGYGDTTPGTPVQLWWTWEGLHQTWTIDRP |
| 96 | MOA2 | *M. procera* | MSTQVSSGQTYKITNVKAGTVIDLSGEDNKSIIGYPYHSGKNQQWTFNWTGKAWTLRSASSGSYLGIEGTPADGTRLVAVNDPFEWHIWRDEANENAFRIFVPFTNYNLDLSGYGDTTPGTPVQLWWTWEGLHQTWTIDRP |
| 97 | MOA3 | *M. procera* | MSTQVSSGQTYKITNVKAGTVIDLSGEDNKSIIGYPYHSGKNQQWTFNWTGKAWTLRSASSGSYLGIEGTPADGTRLVAVNDPFEWHIWRDEANENAFRIFVPFTNYNLDLSGYGDTTPGTPVQLWWTWEGLHQTWTIDRP |
| 98 | PCL1 | *Pleurotus cornucopiae* | MSYTIKVRVYQTNPNAFFRIVEQGVWHYANGGTWSDKDGVLTLTMGGSGTSGMLRFMTEQGKEAFFIAMGVHNYKRWVDIVTGLANDVTCVRALPEYYGDKAERAQSREAQRITQSVLNIDRRNISATYSVAEGNNLELNIVIG |
| 99 | PCL2 | *P. cornucopiae* | MSYTIKVRVFQTNPNAFFRIVEQGVWHYANGGTWSDKDGVLTLTMGGSGTSGMLRFMTEQGKEAFFIAMGVHNYKRWVDIVTGLADDVTCVRALPEYYDDKSERARSREAQRITQSVLNIDRRNISATYSVAEGNNLELNIVIG |
| 100 | POL3 | *P. ostreatus* | MSQIHTVPHALPDFLNARVLDRNVRLRTHAAADEFGFSTKKSKAFGPHRGEAFDEGATWFPLKVVHFTTSNDAISSFRALYRKGATAVYGKVQGGGDLKIVLSSGEYITGIAGTYTDKVTSVQITTSKTVYQSGVKSGESFRFEVPSDHHVIGFHGYADDHIRSLGVSYSRRLASVAPVVTSSTLDDQPQLQPFYSSLCLDDPTRVQWAANNMEAIQQKREQAKADLKPIYTNISDNGENAWVLVESAGQRYYVSYTKSATVWVYVQDQPAQSSGSPDSNPPTTKTCTISLGSYSENSNLLGVSGYVWSNIPVSAPSDTIALAFVNFMKWLVYDGVTWDMAYAATQLSEAMSAVGLQDLAKLIPQSVSKIGGRVQFHESIPTLVVAGVLAVALVFAVLAFAGVVFKEYLLTVSVFNFDSSSIWKSLGWYGDNAELTNGQWENQSLGSFVSSGTALMPPGFSPAKPQNCVVNYVSIVIRNKSEFLQGLGAAMLFSDTTQGATLKYVVHRVESNAIAVKSIPGDPATFDLQEFYKDADSWVQAKSTSTQQGSLTISAYTPFLNGAPDDEYEYLVNIGLPSPV |
| 101 | POL4 | *P. ostreatus* | MSRLPSDFPEMPRSVGGQTLPNFVNAKIIERNVKMPVVNLPHKFDFSIKKSDAFGANTGKAFDEGATWFPLKLVHMTWTANGASSYRGLYGKGATAVYGKVSDGHELKMSLAKDEYITGISGTYTDRITSLSIITTKTTHKAGTDSGESFKFNVPKDHHVVGFHGRADDHIRALGVSYSRRLASIGATRSSLVAADDDQPNIEPFYSNLYLDANTQSQWAKNDMSSIESKRAQAQKDLQPIYDNISKNGETAWTLVEKDGQQYYLSWTQSATMWAYVKDEPQAAGLSAAGDPPKTKTSIISVGSYSMTSNLLGISGYVWKNIPITAIASLIALTFVYFMKGLISDGVAWGIEFAATQLAEAAAAAGVEELAIAIPASVASAGGLIIAGIIGIALFFAVMALADILFRQYFLTVNVFNFDAQHEWKSLGWYSDNADISNGEWKNESIPKFAPAGTGVTPPGFDPVENLENVVTYLSMAFENDSTFLEGLGIGVLISRDDNTEGIAIKYVVHRFSDNDIGIQAITSDPATYDLKGYYNGSWVSGNSTETTLGALRITGHTPYLSGAPNQSYEYNVNIGLPP |
| 102 | POL5 | *P. ostreatus* | MSYKFVVRVYQTNTNAFFKPVEASVYKAGTWANNDGEHILLLGYDTSAAIRLDAGSEHVVVFLGNHDKNVWCDIDTDIGGKSSAQLNMQYYDGQARERDRKKFQKSANITNKKARKFNVELVGDPSSHLLTANIIIH |
| 103 | POL1 | *P. ostreatus* | MSYKFVVRVYQTNTNAFFKPVEASVYKAGTWANNDGEHILLLGYDTSAAIRLDAGSEHVVVFLGNHDKNVWCDIDTDIGGKSSAQLNMQYYDGQARERDRKKFQKSANITNKKARKFNVELVGDPSSHLLTANIIIH |
| 104 | POL6 | *P. ostreatus* | MAYKFTVRVYQTNTNAYFTPIETSVHDAGYWSNTDGQYTLMMGFDTSGAIRLHSGGENVVVFLGNHDKKVWCDIDTDIEGNTAAKLNAEYYDGKARERDRKKHTKSATVVNKKTRRFSLELEGDRNQFVANIIIQ |
| 105 | POL7 | *P. ostreatus* | MAYKFTVRVYQTNTNAYFTPIETSVHDAGYWSNTDGQYTLMTGFDTSGAIRLHSGGENVVVFLGNHDKKVWCDIDTDIEGNTAAKLNAEYYDGKARERDRKKYAKSATVVNKKTRRFSMELEGDRNQFVANIIIQ |
| 106 | POL8 | *P. ostreatus* | MAYKFTVRVYQTNTNAYFTPIETSVHDAGYWSNTDGQYTLMTGFDTSGAIRLHSGGENVVVFLGNHDKKVWCDIDTDIEGNTAAKLNAEYYDGKARERDRKKYAKSATVVNKKTRRFSLELEGDRNQFVANIIIQ |
| 107 | POL9 | *P. ostreatus* | MSTTSMRVFFQDSSLAIREFRHSTASLWSGGTSGDDITLAGRRRAPLAAKEWTESGMRRMRVYTLDAQNNVREVCCDDEASWVDGHHGPAAAPFSDIATTVYDASQRRIRVYYQLPDRQIQELCNDVEWVSGATMRTLPAGTPSLQPIEGTAIASVGWGDTASDIRNYVQDDNGHICEYSTGSGWGAIRLGVRAKLHSPLAAVTWVDSEGRQIRIYYLDDENIIREYFFTTTKGWYDNPLIGSISTRPVKAAPYSRLSSTASKYWIRVYYQPSGLDAIEELCCDFGDSRGWYNGARLSVTPPSSKPIDVDVNTYTLGWLPYLIDDPLAIDGAGILIGFEDGHLISEDLKHKQAMYLAANTKGNSYTYVYTPNGSIVYRLWKHDHRDDYVRHSQVERSKDAICAGEFRVSQAGHIESVVLMKNDSGEYDKPTGGKCLARISEKLESMGITTNDIDWYWK |
| 108 | POL10 | *P. ostreatus* | MTNLWQPGTYYGPGSEVKYNGVNYRIIQPHSSQGDWTPDVTPALWGRMQGGDNDSHNNNNNNQNNNSQNYQQQQPQQQQHNNYGQSDYKPPQPSEQTGYGGKHPEQTVEVTHEEKKKNWFDLDDDRKKQIEIGGGLLAGAAAIGAGFFAYKHHEKNEEEKKAQAWGAQNWLAEAQSRAEAFKRNGPSTAATWVLTEGKHIPKDAILVGQEKSWNLYICRAFIDGGVQLGKASDAFKKGGVIGYGGDENHVSTYEILLGDMRGLVWVNAHGKLNPDSLGKRPVEGGRENDGTPLYVAKAPYKGAVHPGKASEKLNGAYIPYDGEEKRVEDYQVLCYA |
| 109 | POL11 | *P. ostreatus* | MRIPSTTPQLQVNTFTHPPPWGAYITYPPGAVVTYLATIWRCESSHTSWEEPAGVPSSNLAFWTPIGVLGSNVSTSAQQSSPPPYSQSLNAQSEKVPPYQTFMESSPSHNVGSEKPGFDPGHSGSFSYLSDKAGDPFRTPVISEEENESKHEDNLKDEVRGKRFWRVGGLGIWAYGLDTKEESIRERLWREWSDKHNGDDWIKISRKRTKFYNESGGRHVRPLFTWKLVEKGQRLPVDALPIGNEQDGAVLYAARAWHQGGVHLGKAGHHLHKGASIPYGGGEISFDTFEVFCGPINEPHLVKWMTFPHGQIAHVQGWQPVEGGREKDGRALLLAKGFYDNGQHPGKIIVQDDHACVGYGGGEVWIRPYQILAYANPHRR |
| 110 | POL12 | *P. ostreatus* | MSPHNRRKSVDSSDSSSGSDSDHGGKDSHKKEKKDKKDKDKKDKDKKKDKQSAQAANYGHSSTADIDNRQARLHTSQIMDYEQGPPPAYPPPLPRAPSSHPPSGYRVPLTTDNAFPPPAQAGPAVAHDLDGSPIFIGSAIMDGSVHPCKIGPHLQPYVAVPYGGAEFGHHGRYDLLPFDGASMEWVPSSHGRIPPGRSPVEGGYEEGGEKLYHALGDVNGVKVPGKTGEHLNGANVSFGGAEIAVSQYEILCWR |
| 111 | POL13 | *P. ostreatus* | MPFAESSRDWYLEGNVLHATCQDSEGDWVESSLDLDDVLGNNDGEFEAGAEGFSASATDVRIDVDAGYTILRASLQRADGEWNDATIDLNSFIENNNGQLST |
| 112 | POL14 | *P. ostreatus* | MVLLGDLVVALIAAAAPDSQVCDVGSTATCKITATPRQFQPALLNASKWIWSGENPIPGGSNIISTRPFRKNITAPCGKCAVCATIVVASDDAHTFYVNGVRIGTGAGFRQGQALFVALQPTWNLFAIAGQNLVANSPAGIMASILVHFSDGTSETFVTDESWKTLRAAPPENFQLPSTNDSNWSSAAVQGAYQNSVWGPPVLPPVLPLRGSNWIWTSDNVNGAAPVGSRAFRKTVNQCTKVAVCATVLIAADDRYALYVNGATVGSGSSYTVADAYTIPNLHPTFNTFAINATNGGGPAGVIATIIITYSDGSNETVVTDASWKAIQTIPQGFQLPLIDEFGWESAKIIGAFGVAPWGTGMVIPSA |
| 113 | PoLec2 | *P. ostreatus* | MHDISTYNSSFATIARRVVDLNSGSLVLHCNRVSPFDHRRQVNLLYDVLASLTLTLCRLAVAFAFFGALLVAAAPDRQLCGDAENIADKIPSCKVTAPPGPFQPALFNVSKWIWTGENAVPGGVNPVGVRAFRKNITTPCGKCAVHATIIVASDNTHVFYVNGVEIGAGAGWTKGQVLYAALRPSSNLFAIAGGNIPPGGPAGVIASILIHYADGSTDTFITDETWRTVPGAALPKDFQLPATDDSKWGFAALQGIYAKSRWGAVALPPVLGFNDSKWIWSSDHKNGVAPKGTRAFRKTINECAKVAVSATVLIIVDDSYTLYVNGEKVGTANSYTRSQAWTILNLHPTFNTFAISAGNIGGPAGVLATILITYNDGSNQTVVTDGSWKAIQTVPKGFELPFVNEAEWEDAKVVGPFGVGPWGKGVVIPLA |
| 114 | POL15 | *P. ostreatus* | MAESEDKPSKVGDHTIERTIQLRTHSLYAPYIDQDLQNRWWDFGADSYVNTNKHVRLTRPAPSQMGWLWSRLPLTSTNYIIEVEFKISGESSHLFGDGIAVWIAKERAQPGPIFGNKDKFEGLGIFLDTYANSRHSYSFPHVTATIGDGKTEYDYGNDGDDSSIGGCSANFRRTNVATKLKITYIKEKYLDVKIQYKAWDDWSDCFHVENVTLPSAPFIGFSAMTGDVSDAHDIISVTSSSAILAPPAKPKTKSKSVFPSLRTSDDEDPEGSWFGSMFKIFLFIGVCVGGYYGYLEYKRRQAFAGAGNFGGMGGGGYGGGMYANSKRF |
| 115 | POL16 | *P. ostreatus* | MASRCTTLLLLVLALLSPFAMAESEDKPSKVGDHTIERTIQLRTHSLYAPYIDQDLQNRWWDFGADSYVNTNKHVRLTRPAPSQMGWLWSRLPLTSTNYIIEVEFKISGESSHLFGDGIAVWIAKERAQPGPIFGNKDKFEGLGIFLDTYANSRHSYSFPHVTATIGDGKTEYDYGNDGDDSSIGGCSANFRRTNVATKLKITYIKEKYLDVKIQYKAWDDWSDCFHVENVTLPSAPFIGFSAMTGDVSDAHDIISVTSSSAILAPPAKPKTKSKSVFPSLRTSDDEDPEGSWFGSIFKIFLFIGVCVGGYYGYLEYKRRQAFAGAGNFGGMGGGGYGGGMYANSKRF |
| 116 | POL2 | *P. ostreatus* | AIYALPYAAARLLHSLPEDPAAFPKYRVAFLNALPVLNQTAEKWLRDGLRGGELEFLDSQWSEGDVSETYTSPKEIGSGDTHEEFHTSRHQPWDTPYSLEHMKIGPRDSYICFIPKPLEIPPSVDDEVDSDITTLRSWSLLQPLVGTCLYHRQGWFTYSYCHNKEIRQFKELIQAQPHVSGGYKPEEDPEWESYTLGRAPANPEPGADLTVTEQNALAANLELAKGAGSRYLVQKWSDGTFCDKTGRKREVEVQFHCSMTMTDTILFVKETKTCSYILVINTPRLCGEPGFRSPRDTRSEALIRCRAVVDSPQDFPAFDNKLAESDYPFKLPRQKPVLSPPPTEPKGTKSEAKDPKVSLSTEQVDEMVRKALQTFADNKAAGKTDEPNVHIESLGEDGGFVVEFLDEIPLDNINEADEHIDRIADILRAAGFNIKDTVISHKKDSQGAEDGDERKSKKKAGRGEAEPDPYRDEL |
| 117 | POL17 | *P. ostreatus* | MRSVALSVFLVAAAASAASADEQPKPTFAPSDIKAPFLEQFTDDWSERWTPSEATKKTASGGETWSYVGKWEVEDAEPSVIEGDKGLVAKSKAAHHAISAPFSAPIDFKTEPLVVQYEVKYQKGGNCGGGYLKLLEDGFQTSGKDFSDTTPWVVMFGPDLTCPGTKVHFIFRHKNPKTGEYEEKHLKSAPRPSIEKNTKLYTLVVNPNNTYSVSIDGESLSSGSLLEDFDPAVNPPKEIDDPEDKKPEDWVDEKKIADPDATKPDDWDEDAPYEILDEEATKPEGWLEDEPLTIPDPDAQKPEEWDDEEDGDWVAPTVSNPKCAEAPGCGEWKRPFKANPDYKGKWYAPLIDNPAYKGEWAPKKIANPAYFEDLTPVKSLNKIGGVGIELWTMTEDILFDNIYVGHSVDDAKALAAATFEVKKTLETASQKKEMEEEDEDETPSFKEDPVQFIRSKILTFVDAAKIDPLAAFKTQPETGAALAVALFTIFGMIGATLGLIGPQQKPITKSAKKTDAPTPDSKKTDSAPVAPAGGEKKEEGGAKKRTAAAAK |
| 118 | POL18 | *P. ostreatus* | MRSVALSVFLVAAAASAASADEQPKPTFAPSDIKAPFLEQFTDDWSERWTPSEATKKTASGGETWSYVGKWEVEDAEPSVIEGDKGLVAKSKAAHHAISAPFGAPIDFKTEPLVVQYEVKYQKGGNCGGGYLKLLEDGFQTSGKDFSDTTPWVVMFGPDLTCPGTKVHFIFRHKNPKTGEYEEKHLKSAPRPSIEKNTKLYTLVVNPNNTYSVSIDGESLSSGSLLEDFDPAVNPPKEIDDPEDKKPEEWVDEKKIADPDATKPDDWDEDAPYEILDEEATKPEGWLEDEPLTIPDPDAQKPEEWDDEEDGDWVAPTVSNPKCAEAAGCGEWKRPFKANPDYKGKWYAPLIDNPAYKGEWAPKKIANPAYFEDLTPVKSLNKIGGVGIELWTMTEDILFDNIYVGHSVDDAKALAAATFEVKKTLETASQKKEMEEEDEEETPSFKEDPVQFIRSKILTFVDAAKIDPLAAFKTQPETGAALAVALFTIFGMIGATLGLIGPQQKPITKSAKKTDAPTPDSNKTDSAPVAPAGGEKKEEGGAKKRTAAAAK |
| 119 | POL19 | *P. ostreatus* | SVFSYYGQNSYGATHSDTANWQKALSTYCQDDSIDAFPLAFLHVFFASGGLPSINMANTCNTNDNPVFPGTELPNCSFMAADIQACQAKGKIITLSLGGATGAASFSSDAQAQQFADTIWNLFLGGSSSTRPFGNAVLDGIDLDIEGGGSTGFAAFVNRLRTHTNSASKKYYVTAAPQCPFPDAFLGAVLNAVAFDAVYVQFYNNFCGLTNFNNPNAWNFAQWYERAFFCKKSNGRDNWAKTVSPNRNVKIYIGAPAAPTAAGSGYVDPATLASIAVQTRNQFSSFGGIMLWDASQAFANNRFDRAIKNAIRTSGSGGGTTAPP |
| 120 | POL20 | *P. ostreatus* | MALMTAARWFALLSFVCVLATVSAFSNDRSDNLAVYWGQDSGGNQQRLSFYCDDDTIDAFPLAFLYVFFGKGGKPMLDLSNICSQSGSGSFKGTNLADCSFLASDIRTCQAKGKIVTLSLGGATGKVGFNSDSQARGFARDIWDFFLGGDSNTRPFGSAVLDGIDLDIESGSSAHYAAFVNELRSFMNGSGKRYYITAAPQCPFPDQAIGAALNGASFDAVYVQFYNNFCESSRPKDFNFDTWDNWARKQSPNKNVKVYLGAPGAPSAAGDGYVNINTLINLAKDAQKRYPSFGGVMLWDASEARSNNRYDRAIKQALVQDQPSRLPTTTVPSRQPGTTREPATTRAPATTLAPAPAPSPTTTSFAEPEILRPQRGRVMPQTVIHPNLNSRFFRL |
| 121 | POL21 | *P. ostreatus* | MANVAPGTYYIISRVLSPEGEKLALTFNGLGNVTVTPFTNNSKQAWVVKDYNAQNQMFSPASQSNYQIGMGEGKVLAVYPSGGYTWTAKTSATGVLIADAGGVTNFWGLSNAVVDQNVPFGEGNGSDKQRWILIPA |
| 122 | POL22 | *P. ostreatus* | MVGHTSASASAMPNPGTYYIVNRVLSPAGQKLALTFNGERNTVTVTPLSSFHSQWCIRNYYDGATQSVSPASKSRLEIGMGPGTIRVLPPGAYVWTLRNTPSGATIQDGAVTVFWGLSDANIDQEVAFGDEDERGNQRWILIPV |
| 123 | POL23 | *P. ostreatus* | MWLIEPVECFNDTFTVRNLASGNYLDLTDGSKSDGTSIYCRASTGGSNQKWIIKRDLHQEPRKESLWKMQNEASKTFADLYGGGPHLTPIVGWQGSWTEAESIGRTRQWRFECISQTNDQIRTILKASPHVQQSFASDKLYLISSSQRLYAIWGETGLGSTQSREEIFDSDDFALGKPIFKAEVAKWGNKTFKADGFGILCGMMFGSKTDNNGKEISTAYNWNLEVKDLSKVVFYRPMDGTISYDADGYKAYFGLY |
| 124 | RBL1 | *Russula brevipes* | MVYTITAQIYQTNTNAFFHVVEKTIWGSGHAGTWSEIDGAHVLTIGASGASGCLRFRSDTDEFFIVALGIHEGRRWGDIDTLLGAGDTGIVVHPQYYSDAPNLWPRVQQREAQLTSYHVTSLHNRTFSFIYTVPEGRNLKVAIIIG |
| 125 | RBL3 | *R. brevipes* | MVYTITAQIYQTNTNAFFHVVEKTIWGGGHAGTWSEIDGAHVLTIGASGASGCLRFRSDTDEFFIVALGIHEGRRWGDIDTLLGADDTGIVIHPQYYSDAPNLWPRVQQREAQLTSYHVTSLHNRTFSFIYTVPEGRNLKVAIIIG |
| 126 | RBL2 | *R. brevipes* | MILGHLLLSVLALCSSSVLAVDYDGTRIANRTIERTVQLRTHSLFAPYIDQDLQNRWYDFGADTYVNSNKYIRLTRNVQSQMGWLWSRLPLTSTNFIIEVEFKISGSTSHLFGDGIALWITTERAQPGPVFGSKDHFTGLGIFLDTYKNDLDSDLHFPRVVAMQGDGQTSYDVGKDGMQTMIGDCTEEFRHSNVATKLKVIYVKDTVLDVKIQSRGWEQWNDCFTLKDISLPMNPYIGLTAMTGDVSDAHDIVSVSVFSAVLSAAEAPRDKLQVIHSGAGWFSSFVRFLIFGAVLVGAWYGWKMYGRRYFSGNHYGGGGGGLIWSDSKRF |
| 127 | SCL1 | *Schizophyllum commune* | MSACCNKLAVLGLTFFGLLLGLARAEIKIGNTTIDRTVQLRTHSLYAPYIDQDLQNRWWDFGGDAYINTNKHVRLTRMEPSEMGWLWSRLPLTASNFVIEVEFKIGGSKSHLYGDGIGVWVTKERAVEGPIFGNGDRFEGLGVMLDTYANSRHSYAFPRVSGVILDGKTPYDYANDGDSQSIGGCSANYRRAKVATKLKITYVKEKLLDVKIQYKGWDEWTDCFQVQNVKLPTAPYIGFTAMTGDVADAHDIISVTSYSAVLSSPNSSRDRLGKPRLPNLNKKGTWTGLFFKAFLAAGVCVGAWYGYKQYKRKQRYTGFGSGFGPGVLGGKSGPFGGLYGDAKRF |
| 128 | SCL2 | *S. commune* | MFSYLAVLAVALVPALAAPLDGFVARQTPAGTTLHPNGDLTRCLSAATSVVAGEPGARITIDACDGASPQTWVINNGETTVKYTGANSCVTAGGLSDGARLYLEACIPDQVTQTFYYTNDLRIAVENQGLCVDLPSGDKTPGNALQVWTCTDNNTNQIWST |
| 129 | SCL3 | *S. commune* | MFSLYFLTILPAVLATPIARQATTMPAPVYVHPTGNTNFCLGATGDANGAATTIVDCTTKTDSTPSWILDNDKKLFQLNGTQVCLDAGTNIGDGVPLKVWQCYDNLPQQQWTYNTGDQHFSVGPGAECLDLTNGAFTNGAGTQTWSCISYNTNQKWTTSPTDSGNGGGTTPPPPSGNSQVLHPNGNTGKCLDVQGNVQANGTPVEIYDCNGTGAQQWVLSRGHTSVRLAGTNYCLDAGTNIGNGVGMKIWQCYDGLAQQTWYYTDDNRIAVENKGQCLDLTGARTNNGNIVQTWTCTDGNTNQIWY |
| 130 | SCL4 | *S. commune* | MFSVALLAFLPAILAKPYPRQATTAADGTVYIHPNGNTNFCLGTYGAYDSAPVDIFDCSTKTAATPSWALDSSRRTFQLNGTNFCLDAGTNIGDGVSMKLWQCYDNLPQQQWSYNSGDQHFSVSTGAECLDLRDGALYNGGATQTWECSSPNPNQKWTLTTAGSGTGTSSSASSSASGSSSASGSSSGTASSAAPSQTPTQQGQVLHPNGNSAKCLDVQGNVQANGTPVDIYDCNGTSAQQWVLNRGTTAVRLANTNFCLDAGTNIGNGVGMKLWQCYSGLAQQTWYYTNDNRIAVQGYGQCLDLTGGRLDNGNIVQSWTCTDGNTNQIWN |
| 131 | SCL5 | *S. commune* | MFSIAVFALLPAVLAKPLPRQSTSTPMSTGAVYIHPNGNTNFCLGATGTGNGAPVDIFDCSTKTASSPAWVLNPDRQRFQLDGTQMCLDAGVNIGDGVSMKIWQCYDNLPQQQWTYNNDDQHFSVSSGAECLDLRDGVVANGTPAQTWECSAPNTNQKWTTSASSSNTTTPTPPSTTGQVLHPNGNTAKCLDVKGDVANGTPVDISDCDGASDQKWVLSRGTTSVKVAGTNFCLDAGENIGNRVGMKLWQCYDGLAQQTWYYTDDNRIAVEGRGQCLDLTGGVLDNGNVVQTWVCTDGNTNQIWN |
| 132 | SCL6 | *S. commune* | MRTPNLRQLDIQPTPPALKPNLNLAVMLTVVALALLPAVLSKPLPRQTTTSTSTVYIHPNGNTDFCLGAAGSRNGALVDIFDCAMKSESTPTWTLDTDKKRFQLNGTQMCLDAGADITDGVSMKIWQCYDNLPQQQWSYNTDDQHFAVASGSECLDLHDGHVVDGAYTQTWQCSNDNTNQKWTTSESNVSTTPPTSQAQVFHPKGNTKKCLEVYGNIANGSPIEIADCDGTNHQNWVLSRGSTVVKLAGTNFCLDAGEDIRNGATMKLWQCYEGLAQQTFYYTDDNRIAVEGRGQCLDLTGGILEDGNVMQTWACTAGNRNQIWD |
| 133 | SCL7 | *S. commune* | MGIDQGVYLITNAGSGTAATLKDGYVKGWERIKGKEQLNQLWFVKSVKTSASDPGYAAFNLATNKVLDLEKGLADRGTPILGWDYHEGDNQHWVIKKQADGKYYKIQSVKTQTFVDLNNGGKDNGTKIQGWVGSWDETNSHQRWVFNQFSATGSRIKSILDTHPKIHGKVIVEWPDRIYFTPDQYIFEAIWAKTGLADIKPRDPLFQSEAYEKVFKGWVVSRAQEIIKVDGFDILVGSLSLVEKSTQKIKVLDVSVRTSDDKSPDLSQIVFFDPESGRTLVDIPEGYEVNSVII |
| 134 | SCL8 | *S. commune* | MTFLSGVYTITNVGTGTALHLDGHPVQGWEKLSGADRLKQLWFLRPTDTSDSGNSDDAKWVALNIARNKCLDVDSSNSANGTEIIGYPYHKTDNQHWIIRHPASSSYWRFQSVETNTFIDLKKGQSGNGTKKSFTGRQIQAVHYKNGKLKGKVIVEYPDRMCVGFPPSAVTADRVGMLRAAPEQTRIRVCDLIEVDLFAPKAPARSVVDVE |
| 135 | SCL9 | *S. commune* | MRPTTLLNLLAFPVIALAASITSGQSYKITNSKGGTVVDLSAADNTSIIGWPYHEGSNQQILVLFVLYTRHHVPAPMPPCPIIPSAATDTNLLHLQWTLDWAGDGWNFRSLSTGKYISLGGADAANGARLVAETDPFTWHIWTDEQVEGAYRHSDASFPRSGLRLHSSVVFIFVPDTHQNFDLYNYGNSTPGTPITTWYTWDGEHQTWQFDEGEQSRPTSSWHLLAP |
| 136 | SCL10 | *S. commune* | MVEIESGRRYYITNAKAGTVIDLNAMDNYTIVGWNNKNGDNQKWELQRSENGWHIKNVGNGTYLGFDGDLHEGTRLTSWRDPCDWQIWKDEQDPSVYRLFIPNTTLNIDLSDHGNAHPGTAITLWGKWEGRHQCWRFDEA |
| 137 | XCL | *Xerocomellus chrysenteron* | MSYSITLRVYQTNRDRGYFSIVEKTVWHFANGGTWSEANGAHTLTQGGSGTSGVLRFLSTKGERITVAVGVHNYKRWCDVVTGLKPDETALVINPQYYNNGGRDYVREKQLAEYSVTSAIGTKVEVVYTVAEGNNLEANVIFS |
