# Supplementary material for: A lectin gene is involved in the defense of Pleurotus ostreatus against the mite predator Tyrophagus putrescentiae
Source: Front Microbiol. 2023 Apr 27;14:1191500. doi: 10.3389/fmicb.2023.1191500 (PMC10174108; doi:10.3389/fmicb.2023.1191500)
Supplement: Supplementary file 3 [file Data_Sheet_1.docx]

Supplementary Material

A lectin gene is involved in the defense of *Pleurotus ostreatus* against the mite predator *Tyrophagus putrescentiae*

Junjie Liu^1†^, Huiping Li^2†^, Xin Luo^2^, Lin Ma^2^, Cuixin Li^1*^, Shaoxuan Qu^1, 2*^

^1^School of Life Sciences, Southwest Forestry University, Yunnan, China

^2^Institute of Vegetable Crops, Jiangsu Academy of Agricultural Sciences, China

*** Correspondence:** Shaoxuan Qu: qusx@jaas.ac.cn; Cuixin Li: 354802569@qq.com

# Supplementary Tables

Table S1: Primers used in the qRT-PCR reactions of gene expressions in this study;

Table S2: The sequences of all Lectin proteins in this study
